# Control of Stochastic Gene Expression by Host Factors at the HIV Promoter

**DOI:** 10.1371/journal.ppat.1000260

**Published:** 2009-01-09

**Authors:** John C. Burnett, Kathryn Miller-Jensen, Priya S. Shah, Adam P. Arkin, David V. Schaffer

**Affiliations:** 1 Department of Chemical Engineering and the Helen Wills Neuroscience Institute, University of California Berkeley, Berkeley, California, United States of America; 2 Department of Bioengineering, University of California Berkeley, Berkeley, California, United States of America; 3 Physical Biosciences Division, Lawrence Berkeley National Laboratory, Berkeley, California, United States of America; Aaron Diamond AIDS Research Center, Howard Hughes Medical Institute, United States of America

## Abstract

The HIV promoter within the viral long terminal repeat (LTR) orchestrates many aspects of the viral life cycle, from the dynamics of viral gene expression and replication to the establishment of a latent state. In particular, after viral integration into the host genome, stochastic fluctuations in viral gene expression amplified by the Tat positive feedback loop can contribute to the formation of either a productive, transactivated state or an inactive state. In a significant fraction of cells harboring an integrated copy of the HIV-1 model provirus (*LTR-GFP-IRES-Tat*), this bimodal gene expression profile is dynamic, as cells spontaneously and continuously flip between active (Bright) and inactive (Off) expression modes. Furthermore, these switching dynamics may contribute to the establishment and maintenance of proviral latency, because after viral integration long delays in gene expression can occur before viral transactivation. The HIV-1 promoter contains *cis*-acting Sp1 and NF-κB elements that regulate gene expression via the recruitment of both activating and repressing complexes. We hypothesized that interplay in the recruitment of such positive and negative factors could modulate the stability of the Bright and Off modes and thereby alter the sensitivity of viral gene expression to stochastic fluctuations in the Tat feedback loop. Using model lentivirus variants with mutations introduced in the Sp1 and NF-κB elements, we employed flow cytometry, mRNA quantification, pharmacological perturbations, and chromatin immunoprecipitation to reveal significant functional differences in contributions of each site to viral gene regulation. Specifically, the Sp1 sites apparently stabilize both the Bright and the Off states, such that their mutation promotes noisy gene expression and reduction in the regulation of histone acetylation and deacetylation. Furthermore, the NF-κB sites exhibit distinct properties, with κB site I serving a stronger activating role than κB site II. Moreover, Sp1 site III plays a particularly important role in the recruitment of both p300 and RelA to the promoter. Finally, analysis of 362 clonal cell populations infected with the viral variants revealed that mutations in any of the Sp1 sites yield a 6-fold higher frequency of clonal bifurcation compared to that of the wild-type promoter. Thus, each Sp1 and NF-κB site differentially contributes to the regulation of viral gene expression, and Sp1 sites functionally “dampen” transcriptional noise and thereby modulate the frequency and maintenance of this model of viral latency. These results may have biomedical implications for the treatment of HIV latency.

## Author Summary

After HIV genome integration into the host chromosome, the viral promoter coordinates a complex set of inputs to control the establishment of viral latency, the onset of viral gene expression, and the ensuing gene expression levels. Among these inputs are chromatin structure at the site of integration, host transcription factors, and the virally encoded transcriptional regulator Tat. Importantly, transcriptional noise from host and viral transcriptional regulators may play a critical role in the decision between replication versus latency, because stochastic fluctuations in gene expression are amplified by a Tat-mediated positive transcriptional feedback loop. To evaluate the individual contributions of key transcription factor binding elements in gene expression dynamics, we employ model HIV viruses with mutations introduced into numerous promoter elements. Extensive analysis of gene expression dynamics and transcription factor recruitment to the viral promoter reveals that each site differentially contributes to viral gene expression and to the establishment of a low expression state that may contribute to viral latency. This systems-level approach elucidates the synergistic contributions of host and viral factors to the dynamics, magnitudes, and stochastic effects in viral gene expression, as well as provides insights into mechanisms that contribute to proviral latency.

## Introduction

HIV-1 can establish rare, latent infections in cells, and the resulting viral reservoirs represent the most significant barrier to elimination of virus from a patient since they persist for decades and can reactivate at any time [1]. After HIV enters a cell, it integrates its genetic material into the host genome and utilizes host cell transcriptional machinery to regulate its gene expression. Briefly, initial expression from the HIV long terminal repeat (LTR) promoter is hindered by stalling of RNA polymerase II (RNAPII) [2], which results in a high frequency of abortive transcripts [3]. However, a low leaky or basal transcription generates a small fraction of fully elongated transcripts that yield viral mRNA encoding a positive regulator, the transcriptional activator (Tat) [4]. Tat binds to cyclin T1, a unit of the endogenous positive transcriptional elongation factor B (P-TEFb) [5],[6], and the Tat:P-TEFb complex binds to an RNA motif in stalled HIV transcripts known as the transactivation response element (TAR) [7]. In this complex, P-TEFb phosphorylates the C-terminal domain of RNAPII, thereby enhancing its processivity and enabling the efficient generation of fully elongated transcripts [2]. The net result is a strong positive feedback loop of Tat-mediated transactivation that amplifies viral transcriptional elongation nearly 100-fold [8].

We previously explored whether stochastic delays in the onset of HIV-1 Tat expression contribute to the formation of latent viral infections [9]. Genetic noise is an inherent and significant process in gene expression in bacteria [10],[11], yeast [12]–[15], and mammals [9],[16],[17]. In particular, stochastic effects most commonly become important in slow chemical reactions or with low concentrations of chemical species, both of which apply early in HIV gene expression when basal expression and Tat concentrations are low. Using a lentiviral model of the Tat-mediated positive feedback loop (*LTR-GFP-IRES-Tat*, or *LGIT)*, we have demonstrated that random fluctuations in Tat levels could result in clonal cell populations that exhibited two distinct viral gene expression levels, Off and Bright—behavior we refer to as phenotypic bifurcation (PheB) [9]. Such bifurcating clonal populations, expanded from single cells each harboring a single viral integration position, exhibit dynamic gene expression behavior, with cells continuously switching between the two modes of gene expression. Moreover, integrated provirus can remain Off for extended periods of time before switching to a Bright expression level, suggesting that long delays in transactivation could contribute to postintegration viral latency [18],[19]. Here, we expand upon this work to study how host transcription factor binding sites at the HIV-1 LTR contribute to both the level of viral gene expression and noise in that gene expression, with a focus on potential implications for the establishment and persistence of viral latency.

**Figure 1:**
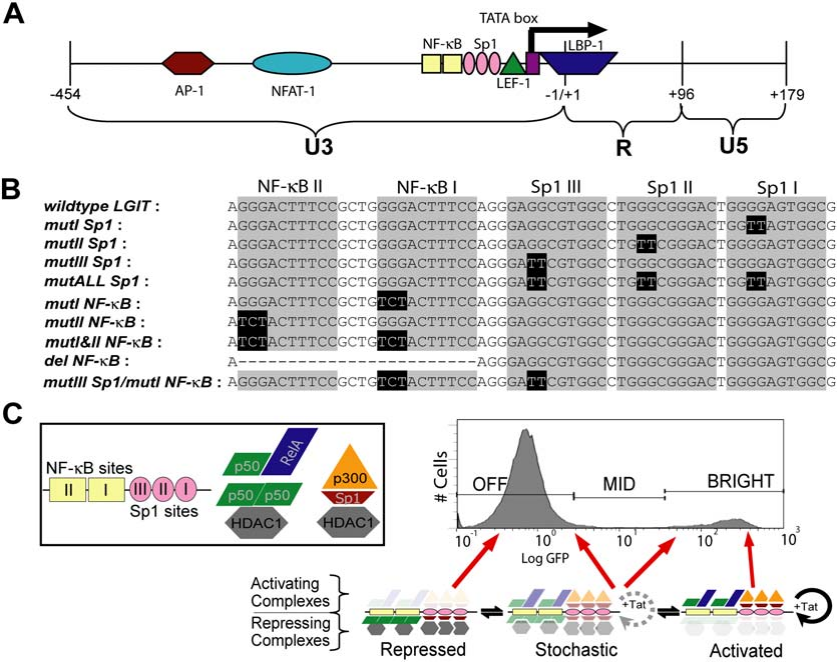
Architecture of Sp1 and κB Regulatory Elements within HIV-1 LTR. (A) Schematic representing the U3, R, and U5 regions of the HIV LTR. Several important transcriptional elements within the U3 region are shown, including the TATA box (−27/−23) and binding elements Sp1 (−55/−46, −66/−57, and −77/−68), κB (−90/−81 and −104/−95), LBP-1 (−16/+27), LEF-1 (−37/−51), NFAT-1 (−254/−216), and AP-1 (−247/−222). (B) Inactivating point mutations in the Sp1 and κB sites were engineered into the *LGIT* lentiviral plasmid. Mutation sites for κB [50] and Sp1 [48],[49] were previously described, and primer sequences are supplied in Table S1. Infections of *LGIT* and mutant lentivirus are detailed in Materials and Methods. (C) A sample bifurcating clonal population of *LGIT*-infected Jurkats. Gene expression of GFP and Tat is amplified by Tat-transactivation, and the two modes of fluorescence (Off and Bright) correspond to the two states in this genetic circuit (Off and On). We hypothesize that transcriptional bimodality is regulated by repressing and activating complexes, which stabilize Off and Bright modes, respectively. These factors may include repressing histone deacetylase (HDACs, including HDAC1) complexes−recruited by p50-p50 homodimer (at κB sites) [3] and Sp1 protein (at Sp1 sites)−and activating histone acetyltransferases (HATs, including p300)−recruited in conjunction with p50-RelA heterodimer (κB sites) and Sp1 protein (at Sp1 sites). The largely unstable Mid region, which may result from stochastic fluctuations in Tat and switching between Off and Bright states, is regulated by dynamic interplay between repressing and activating complexes. See Figure S6 for further detail.

Following preferential HIV-1 integration into regions of active chromatin [9],[18],[20],[21], transcription factor binding sites in the LTR recruit activating and repressing host cell transcription factors and thereby likely influence the basal viral gene expression, the maximal inducible rate of viral expression, and the dynamics of switching between these two states. In particular, binding sites for NF-κB, Sp1, YY1/LBP-1, AP-1, and other factors recruit chromatin modifying complexes to the HIV promoter (Figure 1A) [22],[23]. Activating complexes may recruit histone acetyltransferases (HATs) and thus contribute to stabilizing the transcriptionally active state of HIV in either a Tat- dependent or independent manner [24],[25]. Alternatively, numerous repressing complexes may recruit histone deacetylases (HDACs) that stabilize the transcriptionally inactive mode by chromatin deacetylation or via competition with activating complexes [3],[22].

In particular, the prototypical HIV clade B promoter contains two κB-binding sites and three tandem Sp1-binding sites (Figure 1A and 1B), all of which have the potential to recruit either repressing or activating complexes (Figure 1C). For example, the NF-κB p50-p50 homodimer complex binds to the κB binding sites and can recruit the repressive HDAC1 and HDAC3 factors [3],[26]. Alternatively, binding of the activating NF-κB p50-RelA heterodimer [27] enables interaction with p300 [28],[29], a HAT that is required for full Tat activity [24],[30]. The p50-RelA heterodimer can also interact with P-TEFb [31] and thereby aid RNAPII processivity [32],[33]. Similarly, Sp1 can interact with both HDACs and HATs [34],[35], and thus may mediate both repressing and activating transcriptional mechanisms.

Modulation of HIV gene expression with cytokines and other pharmacological agents that function via NF-κB or Sp1 dependent mechanisms further demonstrates the importance of these sites to promoter regulation. For example, tumor necrosis factor alpha (TNF-α) activates HIV transcription by increasing the nuclear concentration of RelA, thereby increasing the availability of p50-RelA to bind κB sites [36]. In addition, trichostatin A (TSA) activates transcription by inhibiting class I and II HDACs, which otherwise repress HIV gene expression by maintaining chromatin deacetylation [37],[38]. Since both Sp1 and κB sites facilitate recruitment of class I HDACs [3],[34],[39], both NF-κB- and Sp1-mediated repression are targets for TSA activation.

A number of important studies demonstrate that the deletion or mutation of any of the Sp1 or κB elements compromises the rates of gene expression and/or viral replication [40]–[45], though the effects of mutations or deletions on the establishment of latency were not explored. Moreover, although κB sites have been demonstrated to play important roles in both HIV activation and proviral latency [3],[32],[33],[46],[47], the interplay between multiple transcription factor binding sites, gene expression noise [9], and the choice between transcriptional activation and viral replication vs. genetic silencing and latency have not been examined. As we hypothesize that PheB integrants are likely poised at the edge between repressive and activating mechanisms, these proviruses may be highly sensitive to other sources of noise, including the dynamic competition between the recruitment of repressing and activating complexes at the Sp1 and κB sites (Figure 1C).

Here, we examine the roles of the κB and Sp1 elements in the context of a model of postintegration HIV latency to dissect the contributions of each site to gene expression dynamics, transcriptional activation and repression, noise in gene expression, and potentially proviral latency. Using gene expression analysis, pharmacological perturbations, chromatin immunoprecipitation, and analysis of transcriptional initiation and elongation, we demonstrate that each Sp1 site plays a significant role in the persistence of both active and inactive expression states. Furthermore, the two κB sites differentially recruit transcriptional regulators, where κB site I contributes more to transcriptional activation through the recruitment of p50-RelA heterodimer, while κB site II has a bias for the repressing p50-p50 complex. Interestingly, these sites play unique, and at times synergistic, roles in the transcriptional regulation events that underlie gene expression noise and potentially clinical HIV latency.

## Results

### Generation and analysis of lentivirus with Sp1 and κB mutations

Inactivating point mutations [48]–[50] were introduced into each of the Sp1 and κB sites within the LTR of the *LGIT* virus plasmid (Figure 1B). These mutant versions of *LGIT* include an inactivating mutation of Sp1 site I (*mutI Sp1*), Sp1 site II (*mutII Sp1*), Sp1 site III (*mutIII Sp1*), all Sp1 sites (*mutALL Sp1*), κB site I (*mutI NF-κB*), κB site II (*mutII NF-κB*), a combination of κB sites I and II (*mutI&II NF-κB*), a full deletion of both κB sites (*del NF-κB*), and a combination of Sp1 site III and κB site I (*mutIII Sp1/mutI NF-κB*). After viral production, Jurkat cells were infected at a low multiplicity of infection (MOI ∼0.05–0.10), a level demonstrated to yield a polyclonal population of infected cells with a broad range of single viral integration sites per cell [9]. Viral LTR expression was monitored by flow cytometry for 21 days following the initial infection. Gene expression was detectable 48 hours post-infection, the first time point analyzed, and progressively increased over the course of a week. This timing is consistent with *in vivo* reports that reveal that viral production initiates approximately two days after infection following the viral “eclipse phase” [51]. Histograms for *LGIT* and mutant versions revealed a Bright, transactivated population and an Off population that included infected, inactive cells in addition to a larger population of uninfected cells (Figure S1B). However, for two *LGIT* variants, *mutALL Sp1* and *mutIII Sp1/mutI NF-κB*, a Bright population of cells was not detected (Figure S1B), and these two mutant combinations were not further studied.

### Mutants demonstrate activating roles for each Sp1 site

**Figure 2:**
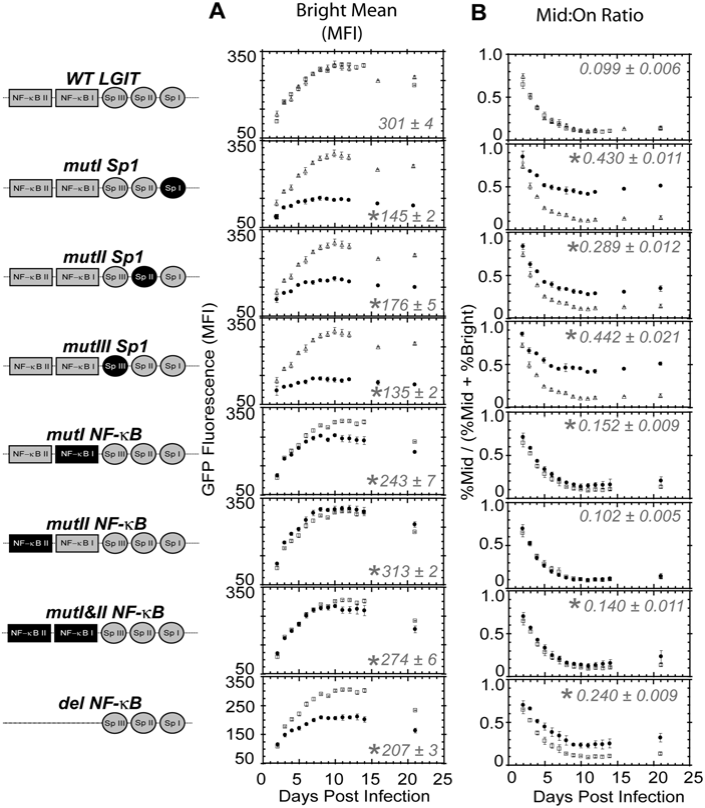
Sp1 and κB Sites Regulate Off and Bright Dynamics. (A) Jurkat cells were infected with *LGIT* and corresponding Sp1 and κB mutants at low MOI (∼0.05–0.10) in biological triplicate (for WT *LGIT* and Sp1 mutants) or biological quadruplicate (for WT *LGIT* and κB mutants), and data are the averages of these replicates at each day of the 21-day time course. Shown are the mean of the Bright peak positions (as illustrated in Figure 1C) from the GFP histograms for all time points, as measured by flow cytometry in units of mean fluorescence intensity (MFI). The results from WT *LGIT* control for two separate experiments (open square or triangle points) are shown together. Data for each mutant (solid circle points) are shown with the corresponding WT *LGIT* control. Error bars are the standard deviation of the biological quadruplicate or triplicate measurements. Statistically significant differences from WT *LGIT* are denoted by single asterisks (*, p<0.01). The steady state Bright Mean values at 10 days after infection are shown within each panel. Two *LGIT* variants (*mutALL Sp1* and *mutIII Sp1/mutI NF-κB*) failed to generate a GFP^+^ population of cells after infection at low MOI (∼0.05–0.10) and were thus omitted from this study (see Figure S1B). Further details of data analyses are in available in Materials and Methods. (B) The same experiment as in (A), but depicting the fraction of infected and GFP^+^ cells persisting in the Mid region (Mid:On ratio), in which “On” is the sum of “Mid” and “Bright” regions (Figure 1C), for the duration of the time course. Error bars are the standard deviation of the biological quadruplicate or triplicate measurements. Statistically significant differences from WT *LGIT* are denoted by single asterisks (*, p<0.01). The “steady state” Mid:On values at 10 days after infection are shown within each panel.

For the 21-day time course experiments, heat maps depicting the GFP intensity distribution of the infected cell populations indicate that mutations in the Sp1 sites substantially impact GFP expression (Figure S1A). The WT and mutant *LGIT* variants exhibited a similar temporal onset of gene expression and reached a maximum in the mean position of their bright peaks (Bright Mean)—a metric of gene expression in the Tat feedback loop—10 days after infection (Figure 2A). Importantly, mutation of any of the Sp1 sites (*mutI Sp1*, *mutII Sp1*, and *mutIII Sp1*) resulted in dramatic 40–50% decreases in the Bright Mean levels (Figure 2A). These results indicate that each Sp1 site has a considerable role in the transcriptional activation of the proviral LTR, with Sp1 site III appearing to have a slightly larger contribution than Sp1 site I or II (p<0.05 for each day after day 6).

While the Bright Mean characterizes the strength of Tat transactivation within the positive feedback loop, a smaller, less stable population of *LGIT* cells exhibits intermediate levels of gene expression. We have previously demonstrated that stochastic effects in gene expression are most evident at these intermediate levels of Tat and contribute to switching between Bright and Off modes [9]. Therefore, the fraction of cells that expresses GFP at intermediate or Mid fluorescence levels (i.e., the Mid:On ratio, where On is the sum of Mid and Bright regions, Figure 1C) is a measure of stochastic fluctuations in Tat expression. Mutations that further stabilize the Off or Bright mode would be predicted to result in a lower Mid:On ratio and reduced “flipping” between the two stabilized states. In contrast, mutations that destabilize the Off and Bright modes would yield an increase in the Mid:On ratio, via increasing the rate of flipping between the two “less stable” transcriptional states and thereby creating a noisier promoter. At early times after infection, the Mid:On ratio is high, as the gene expression of infected cells ramps up, but it later settles into an informative steady state value (Figure 2B). Over the three week time course, the Mid:On ratios for each of the Sp1 mutants remain 3- to 4-fold higher than WT. These data indicate that each of the Sp1 sites in the WT promoter may stabilize the Bright and potentially the Off mode, and that a reduction of this stabilization (consistent with the observed decrease in the Bright Mean position, Figure 2A) may increase the rates of switching between Off and Bright expression modes. Thus, based on the Mid:On ratio as a metric for stochastic behavior in the Tat-feedback circuit, the Sp1 sites appear to control promoter noise, with potential implications for viral latency.

### Mutants suggest distinct roles for the two κB sites

In parallel experiments to the *LGIT* Sp1 mutants, mutation of each of the two κB sites in the HIV promoter reveals the roles of each site in stabilizing the Bright modes (Bright Mean) as well as dynamic flipping between modes (Mid:On ratio). Compared to WT *LGIT*, the κB site I mutant (*mutI NF-κB*) exhibited a decrease of the Bright Mean, whereas mutation of κB site II (*mutII NF-κB*) yielded a slight, but statistically significant (p<0.05 at two weeks after infection) increase of the Bright Mean (Figure 2A). Interestingly, these results indicate that the roles of the two κB sites in the HIV promoter are not redundant, with an intact site I serving an activating role and site II a slightly repressing role. Consistent with these observations, the double κB mutant (*mutI&II NF-κB*) exhibited gene expression levels closer to those of the WT promoter than *mutI NF-κB*, indicating that the loss of the repressing site II slightly counteracts the loss of the activating site I. In contrast to *mutI&II NF-κB, del NF-κB* exhibited a severe loss in gene expression, indicating that the complete deletion of the 24 nucleotides encompassing the κB sites had effects beyond the loss of NF-κB binding, perhaps through altered nucleosome spacing [36] or loss of the NFAT1 and GABP transcription factor binding sites at the 3′ ends of the κB sites [52],[53], which were not affected by the individual mutations in *mutI&II NF-κB*. To focus analysis specifically on the roles of κB recruitment, we did not pursue analysis of the variant with full deletion of both κB sites.

In contrast to the Sp1 mutants, mutation of the κB sites had modest effects on the Mid:On ratio compared to the WT LTR (Figure 2B). However, significant differences between κB sites I and II are evident, as *mutII NF-κB* had no change in the Mid:On ratio, but *mutI NF-κB* exhibited a 1.5-fold increase compared to the WT promoter. Thus, the observed decrease in the Bright Mean position of *mutI NF-κB* (Figure 2A) is consistent with destabilization of the Bright mode, resulting in noisier gene expression or an increased Mid:On ratio (Figure 2B).

### Promoter mutations increase the population of “Infected but Off” cells

**Figure 3 ppat-1000260-g003:**
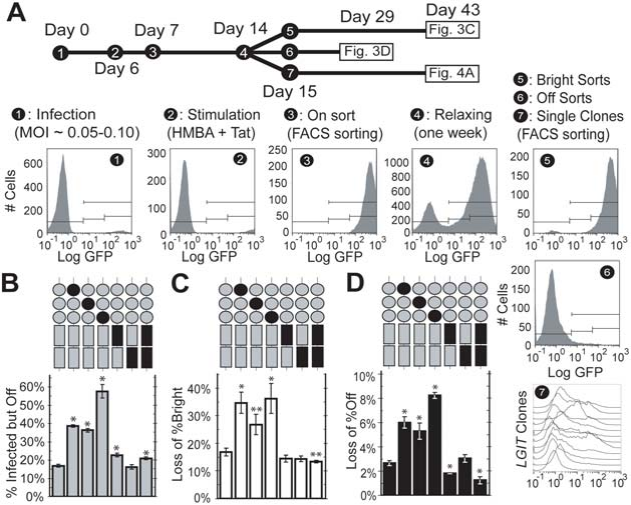
Sp1 Sites Regulate Fraction of Infected but Off Dynamic Switching. (A) Jurkat cells were infected with either *LGIT*, *mutI Sp1*, *mutII Sp1*, *mutIII Sp1*, *mutI NF-κB*, *mutII NF-κB*, or *mutI&II NF-κB* lentivirus at low MOI (∼0.05-0.10) (panel 1). Six days post-infection, *LGIT* gene expression was stimulated with HMBA and exogenous Tat protein (panel 2). Eighteen hours after stimulation, GFP+ cells were sorted with FACS to isolate infected from uninfected cells (panel 3), and cells were then cultured under normal conditions for one week to allow relaxation of expression levels (panel 4). After relaxing into Off and Bright peaks, FACS sorting was used to isolate the polyclonal Bright fraction (panel 5), the polyclonal Off fraction (panel 6), and individual clones (panel 7). (B) Infected but Off cells persist in the Off state in unstimulated conditions. Cells infected with WT *LGIT*, *mutI Sp1*, *mutII Sp1*, *mutIII Sp1*, *mutI NF-κB*, *mutII NF-κB*, and *mutI&II NF-κB* were stimulated with HMBA and exogenous Tat protein to determine the total number of infected cells (Figure 3A, panel 2). Shown are the fractions of infected cells that persist in the Off state (%Infected but Off). These data are calculated by the simple formula: %Off__infected_ = (1−%On__unstimulated_)/(%On__stimulated_). All data are averages of biological triplicates, and error bars are standard deviations. Statistically significant differences from WT *LGIT* are denoted by single asterisks (*, p<0.01). (C) Bright-sorted *LGIT* cells spontaneously inactivate into the Off mode under normal culturing conditions. Bright-sorted populations (Figure 3A, panel 5) were cultured for 14 days after FACS sorting to quantify the stability of the Bright mode. As analyzed by flow cytometry, a fraction of Bright-sorted cells relaxed out of the Bright mode, which is indicated by “Loss of %Bright.” Error bars are standard deviations of triplicate measurements. Statistically significant differences from WT *LGIT* are denoted by single asterisks (*, p<0.01). (D) Off-sorted *LGIT* cells spontaneously activate into the Bright mode under normal culturing conditions. Off-sorted populations (Figure 3A, panel 6) were cultured for 28 days after FACS sorting to quantify the stability of the Off mode. As analyzed by flow cytometry, a fraction of Off-sorted cells activated from the Off mode, which is indicated by “Loss of %Off.” Error bars are standard deviations of triplicate measurements, and statistically significant differences from WT *LGIT* are denoted by single asterisks (*, p<0.01).

Infecting cells at an MOI of 0.05–0.10 results in approximately 90–95% of cells being uninfected (Figure 3A, panel 1) as predicted by a Poisson distribution. However, a fraction of the infected cells may conceivably persist in the Off mode and thus be indistinguishable from the uninfected cells by flow cytometry. This fraction of “Infected but Off” cells provides additional insights into the relative stability of the Off and Bright modes for the different mutants. Specifically, increases in the fraction of Infected but Off cells suggest an increase in the stability of the Off mode or a decrease in the stability of the Bright mode, impeding cells from undergoing Tat transactivation. To measure the fraction of Infected but Off cells, we stimulated gene expression through simultaneous addition of exogenous Tat [18] and the hybrid polar compound hexamethylene bisacetamide (HMBA), which activates HIV transcription independent of the NF-κB pathway [54]. Six days after infection, cells were treated with 5 mM HMBA and Tat protein (8 µg per 3×10^5^ cells) and incubated for 18 hours (Figure 3A, panel 2). This combined stimulation increased the fraction of the WT *LGIT* infected cells that expressed GFP by 17.0%±0.8% of infected cells, which would otherwise persist in the Off mode (Figure 3B).

Interestingly, all three Sp1 mutants exhibited considerably higher fractions of Infected but Off cells, peaking with *mutIII Sp1* at 57.6%±3.7% (Figure 3B). In addition, mutation of κB site I (in both *mutI NF-κB* and *mutI&II NF-κB*), but not κB site II, resulted in a more modest but significant increase in the fraction of infected cells being Off (Figure 3B). Specifically, *mutII NF-κB* is indistinguishable from WT *LGIT* (p = 0.64), but *mutI NF-κB* exhibited statistically higher fractions of Infected but Off cells (p<0.01). These are consistent with our observations that the two κB sites are functionally different, with κB site I having a stronger activating role (Figure 2A). Collectively, these data indicate that loss of any of the Sp1 sites, and to a lesser degree κB site I, destabilizes the Bright, transactivated expression state.

### Mutation of Sp1 sites destabilizes both transactivated (Bright) and latent (Off) gene expression modes

To examine the relative stabilities and switching dynamics of the Bright and Off modes of expression, we sorted pure populations of infected cells that had persisted in the Bright mode (Bright sort, Figure 3A, panel 5) or relaxed into the Off mode (Off sort, Figure 3A, panel 6). The polyclonal Bright and Off sorts are phenotypically homogeneous populations of singly-integrated cells that represent a wide range (>10^5^) of integration positions. The distribution of viral gene expression in Bright and Off modes was dynamic, and the stability of the Bright mode of the bimodal distribution of *LGIT* was determined by measuring the spontaneous inactivation or relaxation of Bright-sorted cells (Figure 3A, panel 5). Fourteen days after sorting the Bright population, the frequencies of spontaneous inactivation (%Loss of Bright) for each of the three individual Sp1 mutations (*mutI Sp1*, *mutII Sp1*, and *mutIII Sp1*) increased significantly compared to WT *LGIT* (Figure 3C). Consistent with previous findings (Figures 2A and 3B), this trend again indicates that each Sp1 site may contribute to the stability of the Bright mode. In contrast to the Sp1 mutants, the frequencies of spontaneous inactivation for the κB site mutants (*mutI NF-κB* and *mutII NF-κB*, and *mutI&II NF-κB*) were unchanged compared to WT *LGIT* (Figure 3C, p = 0.20 and 0.15, respectively), suggesting that the κB sites play a comparatively smaller role in the stability of the Bright mode.

To examine the stability of the Off mode we measured the spontaneous initiation of GFP expression from the Off-sorted cells (Figure 3A, panel 6), which we refer to as spontaneous activation. After 28 days of culturing the Off-sorted cells for WT *LGIT*, fewer than 3% of these cells activated out of the Off region (%Loss of Off), which demonstrates the stability of its Off mode. However, mutation of any of the three Sp1 sites resulted in dramatic increases (2- to 3-fold) in the rates of spontaneous activation compared to WT *LGIT* (Figure 3D), indicating that each of these three mutants has a destabilized Off mode. This result implies that in the Off state, Sp1 sites may be involved in a repressive mechanism, such as recruitment of HDAC complexes by individual Sp1 proteins [34],[39],[55]. This observation is surprising in light of earlier results suggesting that each Sp1 site is required for strong activation (Figure 3B and 3C). Each of the Sp1 sites may thus serve a repressing role in the Off mode and an activating role in the Bright mode, and the dynamic interplay between these roles may contribute to transcriptional noise and stochastic switching.

Analysis of the Off mode also revealed a reduction in spontaneous activation for *mutI NF-κB* and *mutI&II NF-κB* (by approximately 30% and 50%, respectively) relative to WT (Figure 3D), consistent with earlier observations that κB site I is important for the recruitment of an activating complex (Figures 2A and 3B). By contrast, κB site II did not affect spontaneous activation, as *LGIT* and *mutII NF-κB* are statistically indistinguishable (Figure 3D, p = 0.31), suggesting that in the Off state the second κB site does not recruit the same activating complex as the proximal site. Again, the roles of the two κB sites significantly differ (as in Figures 2 and 3B), with κB site I exhibiting a greater activating role than κB site II.

### Promoter mutations increase the frequency of phenotypic bifurcation

In addition to regulating the overall dynamics and steady states of viral gene expression, the individual Sp1 and κB elements may influence stochastic aspects of viral gene expression and thereby affect viral latency. We hypothesized that the dynamic balance in the recruitment of repressing and activating factors to individual promoter sites (Figure 1C) modulates the stabilities of the Off and Bright modes of gene expression, and mutation of these sites would therefore impact the frequency of phenotypic bifurcation (PheB), singly infected clonal cell populations that split into Off and Bright gene expression modes [9].

To analyze the role of individual transcription factor binding sites in the bifurcation phenotype, we isolated 362 individual clones from WT and mutant *LGIT* populations. Six days after infection, *LGIT* (and mutant) infected cells from Figure 2 were transiently stimulated with HMBA/Tat, and the infected (On) populations were isolated using fluorescence activated cell sorting (FACS) (Figure 3A, panel 3). These polyclonal populations were allowed to relax for one week, and single cells were then sorted from the Mid region, expanded, and analyzed by flow cytometry for heterogeneous expression and Phenotypic Bifurcation (PheB) in gene expression levels (Figure 3A, panel 7).

**Figure 4:**
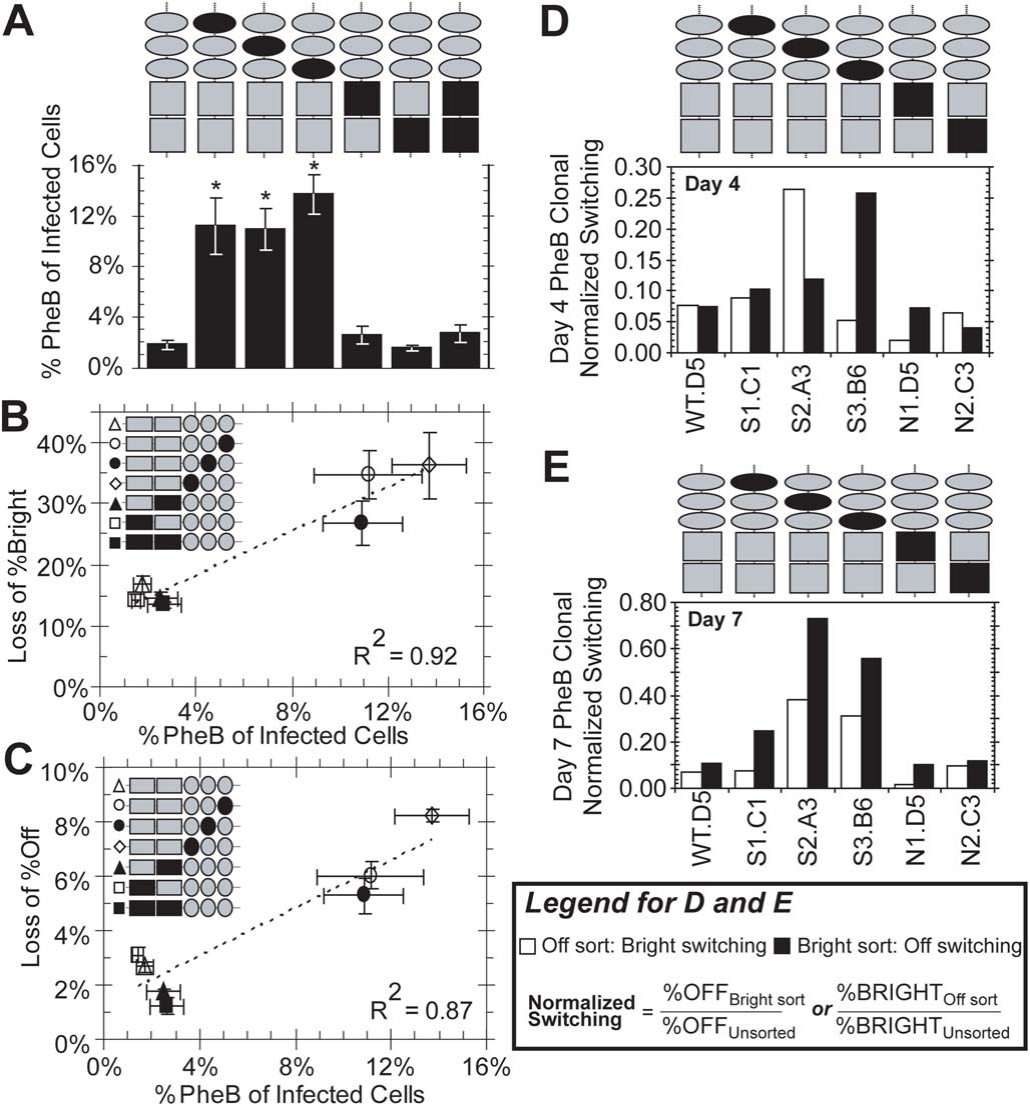
Sp1 Sites Regulate Phenotypic Bifurcation and Transcriptional Dynamics. (A) Clonal populations phenotypically bifurcate (PheB) into Off and Bright modes. Clonal FACS-sorting was performed to isolate single cells from *LGIT*, *mutI Sp1*, *mutII Sp1*, *mutIII Sp1*, *mutI NF-κB*, *mutII NF-κB*, and *mutI&II NF-κB* infected populations (Figure 3A, panel 7). Each single cell was expanded as a clonal population to achieve least 10^6^ cells and analyzed by flow cytometry to measure GFP expression. PheB was defined as a clonal population having more than 0.5% of cells in each of the “Off” and “Bright” gates after four weeks of expansion in normal culturing conditions. In total, 362 *LGIT* and *LGIT* mutant clones were sorted, expanded, and analyzed. Of these, 190 exhibited PheB behavior in GFP gene expression. To determine statistical variance for each mutant, qualification for each clone (either PheB or non-PheB) from each mutant were randomly placed into one of three bins, and the error bars represent the standard deviations for the three bins. (B) Correlation of spontaneous inactivation (Figure 3C) and Phenotypic Bifurcation (A). Together, these data show the correlation between the stability of the Bright mode (Loss of %Bright) and the degree of transcriptional noise (%PheB). (C) Correlation of spontaneous activation (Figure 3D) and Phenotypic Bifurcation (A). Similarly to (B), these data show the correlation between the stability of the Off mode (Loss of %Off) and the degree of transcriptional noise (%PheB). (D) Off and Bright fractions of one phenotypically bifurcating (PheB) clone from each *LGIT* variant were isolated with FACS (Figure S2). Four days after sorting, Off and Bright sorts were analyzed by flow cytometry to measure the extent of dynamic switching. Each “normalized switching” value is the fraction of cells that have switched into the specified region divided by the fraction of cells in that region for the unsorted population. White bars indicate the switching of Off sorts into the Bright region, and black bars indicate the switching of Bright sorts into the Off region. (E) The same as in (D) with flow cytometry analyses performed seven days after FACS sorting. White bars indicate the switching of Off sorts into the Bright region, and black bars indicate the switching of Bright sorts into the Off region. Histograms are provided in Figure S2.

The frequency of bifurcation for WT *LGIT* was 1.77%±0.35% (Figure 4A), a level consistent with our prior findings [9]. In addition, all κB mutations yielded a PheB-clone frequency statistically indistinguishable from WT *LGIT*. Strikingly, however, all three Sp1 mutants exhibited a greater than 6-fold increase in the frequency of PheB. These results are consistent with the increased Mid:On ratio for Sp1 mutants (Figure 2B) and the increased dynamic switching between Off and Bright sorts (Figure 3C and 3D), and further indicate that the loss of any of the three Sp1 sites increases stochastic flipping between Off and Bright modes.

Mutation of any Sp1 site thus renders the viral promoter both weakly silenced (Off) and weakly transactivated (Bright), resulting in increased rates of spontaneous switching and phenotypic bifurcation. In agreement with this interpretation, there are compelling correlations between the frequency of PheB and the fraction of spontaneous inactivation (Figure 4B) and spontaneous activation (Figure 4C), indicating increased transcriptional noise and stochastic switching between two “less stable” states (Figure 1C). Together, these data reveal that each Sp1 site−and particularly Sp1 site III−plays an important role in the control of stochastic gene expression by regulating the active and inactive gene expression states via the recruitment of activating and repressing factors. This is the first demonstration that specific *cis*-regulatory elements within the HIV promoter contribute to transcriptional stochasticity and implicates the Sp1 sites as significant factors in the establishment and maintenance of proviral latency.

### Switching dynamics of PheB clones for each Sp1 and κB *LGIT* mutant

To further support our hypothesis that the Sp1 mutants have increased switching dynamics, we have examined the switching dynamics of Off and Bright sorts of PheB clones for the *LGIT* variants (Figure S2). We hypothesize that the clonal Off and Bright sorts may exhibit switching dynamics similar to the polyclonal populations (Figure 3C and 3D) and may partially converge back to the original bimodal distribution. Due to the rarity of clonal populations exhibiting PheB for all *LGIT* variants (∼2%–15% depending on mutant, Figure 4A)—and since gene expression profiles widely vary between different PheB clones—isolating and identifying different PheB clones that have identical gene expression profiles was not possible. However, we selected one PheB clone for each *LGIT* mutant that exhibited similar bimodality and isolated the Bright and Off modes using FACS (Figure S2). We have normalized the measured switching effects by the distribution from the unsorted clone, and the resulting “normalized switching” value provides a metric for the convergence to the original bimodal distribution. Values ranging from zero (no switching) to one (complete convergence) enable the evaluation of clonal switching dynamics for each Sp1 or κB mutant.

At four and seven days after FACS sorting, we have measured the GFP distributions for the unsorted, Off-sorted, and Bright-sorted fractions (Figure S2). The Bright fractions for each Sp1 mutant clone (S1.C1, S2. A3, and S3.B6) exhibit increased switching into the Off region (Figure 4D and 4E), which mimic increased spontaneous inactivation in polyclonal Bright sorts (Figure 3C). Similarly, Off-sorted fractions from the clones for *mutII Sp1* (S2.A3) and *mutIII Sp1* (S3.B6) have dramatically enhanced switching into the Bright region seven days after sorting (Figure 4E), which are consistent with spontaneous activation of the polyclonal Off sorts (Figure 3D). In contrast, the Off-sorted fraction from the clone for *mutI NF-κB* (N1.D5) exhibits decreased switching into the Bright region (Figure 4D and 4E), consistent with the observed polyclonal dynamics that this mutant has a stabilized Off mode (Figure 3D). Collectively, clonal and polyclonal switching dynamics reveal destabilization of the Off and Bright modes for the Sp1 mutants (Figure 3C and 3D).

### *mutIII Sp1* is desensitized to TSA and *mutI NF-κB* is resistant to TNF-α induction

To identify mechanistic differences in the roles of individual Sp1 and κB sites, we performed exogenous perturbations on each *LGIT* variant. Two weeks after infection with *LGIT* or mutants (the same unsorted populations analyzed in Figures 2 and 3A, panel 1), cells were stimulated with TNF-α (20 ng/ml) or TSA (400 nM) for 18 hours. The change in Bright Mean after perturbation revealed differential contributions for each site in the Bright mode (histograms in Figure S1B and non-normalized data in Table S4).

**Figure 5:**
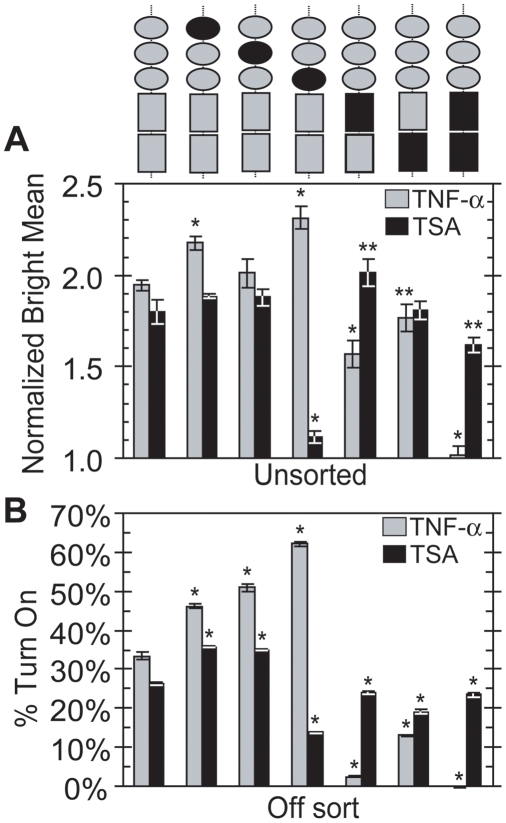
Perturbations of Sp1 and κB Mutants. (A) Stimulation with TNF-α or TSA increases the Bright Mean. Unsorted populations infected with *LGIT*, *mutI Sp1*, *mutII Sp1*, *mutIII Sp1*, *mutI NF-κB*, *mutII NF-κB*, and *mutI&II NF-κB* (same as in Figure 2) were stimulated with TNF-α (gray bars) or TSA (black bars) two weeks after infection. The Bright Mean position of stimulated cells and control (unperturbed) cells was measured by flow cytometry 18 hours after stimulation. Notably, no significant change in the Bright Mean was observed for *mutI&II NF-κB* upon TNF-α stimulation, confirming that the κB mutations abrogate NF-κB-mediated activation. The Normalized Bright Mean for *LGIT* and all *LGIT* variants was normalized by the unstimulated Bright Mean for each corresponding variant (see Figure 2A). Raw data for these measurements are provided in Table S4. All data are averages of biological triplicates, and error bars are standard deviations. Histograms of these perturbations are presented in Figure S1B. Statistically significant differences from WT *LGIT* are denoted by single asterisks (*, p<0.01) and double asterisks (**, p<0.05). (B) Stimulation with TNF-α or TSA activates the infected cells that persist in the Off state in unstimulated conditions. Cells were prepared to isolate the fraction of “Infected but Off” cells by serial FACS sorting (Figure 3A, panel 6). At day 17 post-infection and three days after FACS sorting from the Off region, cells were stimulated with TNF-α or TSA. The data are the fraction of Off-sorted cells that activate into the On region after stimulation. Flow cytometry measurements were performed 18 hours after stimulation. All data are averages of biological triplicates, and error bars are standard deviations. Statistically significant differences from WT *LGIT* are denoted by single asterisks (*, p<0.01).

Although each of the three Sp1 mutants exhibited a lower Bright Mean than WT (Figure 2A), stimulation with TNF-α strongly increased the Bright Mean position of *mutI Sp1*, *mutII Sp1*, and *mutIII Sp1* (Figure 5A, gray bars), confirming that these promoters are susceptible to activation via NF-κB dependent pathways. Furthermore, stimulation with the HDAC inhibitor TSA increased the Bright Mean almost 2-fold in *mutI Sp1* and *mutII Sp1* (Figure 5A, black bars). Since Sp1 has been shown to recruit class I HDACs to the HIV promoter [39], TSA inhibition of these HDACs may shift the chromatin modification balance towards acetylation by HATs. However, *mutIII Sp1* was strikingly insensitive to TSA (Figure 5A), suggesting that this mutant may have minor regulation by HDACs or that it may not have sufficient HAT occupancy to take advantage of HDAC inhibition. Of these two possibilities, the former is consistent with a destabilized Off mode (Figures 3D and 4C), while the latter is consistent with a destabilized Bright mode (Figures 3C and 4B).

Both *mutI NF-κB* and *mutII NF-κB* were activated by TNF-α, though to a lesser extent than the WT promoter. However, *mutI NF-κB* was slightly but significantly less activated than *mutII NF-κB* (p<0.05), consistent with our prior findings that *mutI NF-κB* has lower levels of gene expression than *mutII NF-κB* (Figure 2A). TNF-α induced no relative change in the Bright Mean position for *mutI&II NF-κB*, confirming that this double mutation eliminated NF-κB-mediated activation of the HIV promoter (Figure 5A, gray bars). Stimulation with TSA strongly increased the Bright Mean position for all κB mutants, including *mutI&II NF-κB*, as its effects are not dependent upon NF-κB activation (Figure 5A, black bars). However, in contrast to TNF-α, TSA activated *mutI NF-κB* more strongly than *mutII NF-κB* or WT (p<0.05), suggesting that *mutI NF-κB* may be more heavily repressed by class I HDACs.

### Reactivation of Off sorts to probe latency mechanisms

Using the Off-sorted polyclonal populations (Figure 3A, panel 6) as a model for HIV latency, we examined the stability of the Off mode by measuring the susceptibility of Off-sorted cells to activation by TNF-α and TSA. TNF-α activated approximately 33% of the Off-sorted *LGIT* cells, demonstrating that a large fraction of these “latent” cells is capable of reactivation via a NF-κB-dependent mechanism (Figure 5B, gray bars). Each of the Off-sorted Sp1 mutants responded more strongly to TNF-α induction than WT *LGIT* (Figure 5B). These results are consistent with the previously observed increase in Bright Mean position for these mutants (Figure 5A), suggesting that these mutants are deficient in recruiting RelA under unstimulated conditions.

TSA activates approximately 35% of the Off-sorted, “latent” cells of the *mutI Sp1* and *mutII Sp1* populations (Figure 5B, black bars) but only 13% of *mutIII Sp1* cells, analogous to results in unsorted cells (Figure 5A, black bars). These results suggest that all these mutants are repressed by HDACs in the Off state, but that Sp1 site III is specifically required for an effective response to TSA, possibly because it plays a key role in recruitment of HAT complexes.

In contrast to WT *LGIT* and the corresponding Sp1 mutants, in which at least one-third of the Off cells were activated by TNF-α, both *mutI NF-κB* and *mutI&II NF-κB* were virtually insensitive to TNF-α stimulation, indicating that κB site I is essential for NF-κB-dependent activation of Off cells (Figure 5B, gray bars). However, 13% of *mutII NF-κB* cells responded to TNF-α stimulation (Figure 5B), further demonstrating that when intact, this κB site plays a significant, but weaker, role in NF-κB activation than site I. Finally, all three κB mutants exhibited reduced responses to TSA stimulation compared to WT *LGIT* (Figure 5B, black bars), suggesting that both κB sites have significant but unequal roles in the recruitment of p50-p50 homodimer and HDAC complexes in the latent state.

### Chromatin immunoprecipitation on Off- and Bright-sorted polyclonal populations

The gene expression and perturbation results thus far suggest that individual Sp1 binding sites differentially recruit activating and repressing transcription factors, thereby differentially stabilizing the Off and Bright expression modes and contributing to gene expression noise (Figure 4B and 4C). We used chromatin immunoprecipitation (ChIP) to measure p50, RelA, p300, Sp1, and HDAC1 protein occupancy at the LTR in populations sorted from Off (Figure 3A, panel 6) and Bright (Figure 3A, panel 5) regions. Additionally, we have analyzed Off and Bright sorts for acetylation of lysines 9 and 14 of the tail of histone 3 (AcH3, markers for active chromatin [54]) and trimethylation of lysine 9 (TriMetH3K9, a signature of repressed chromatin [56]). Performing ChIP on Off- and Bright-sorted populations is distinct from recent ChIP analyses on chromatin targets of transfected and/or integrated LTR, which used pharmacological factors including TNF-α, TSA, and phorbol esters to observe occupancy and histone acetylation patterns in the stimulated or unstimulated wild type LTR [3],[32],[46],[47],[57],[58]. Our work focuses instead on analyzing differences in the occupancies of chromatin regulators and transcription factors within Off and Bright modes of integrated viral mutants in unstimulated conditions. Such quantitative differences in LTR occupancy between two coexisting cell populations may influence and reflect the fate of the provirus towards transcriptional activation or repression and latency.

### Recruitment of RelA in the Off mode is mediated by κB site I and Sp1 site III

**Figure 6:**
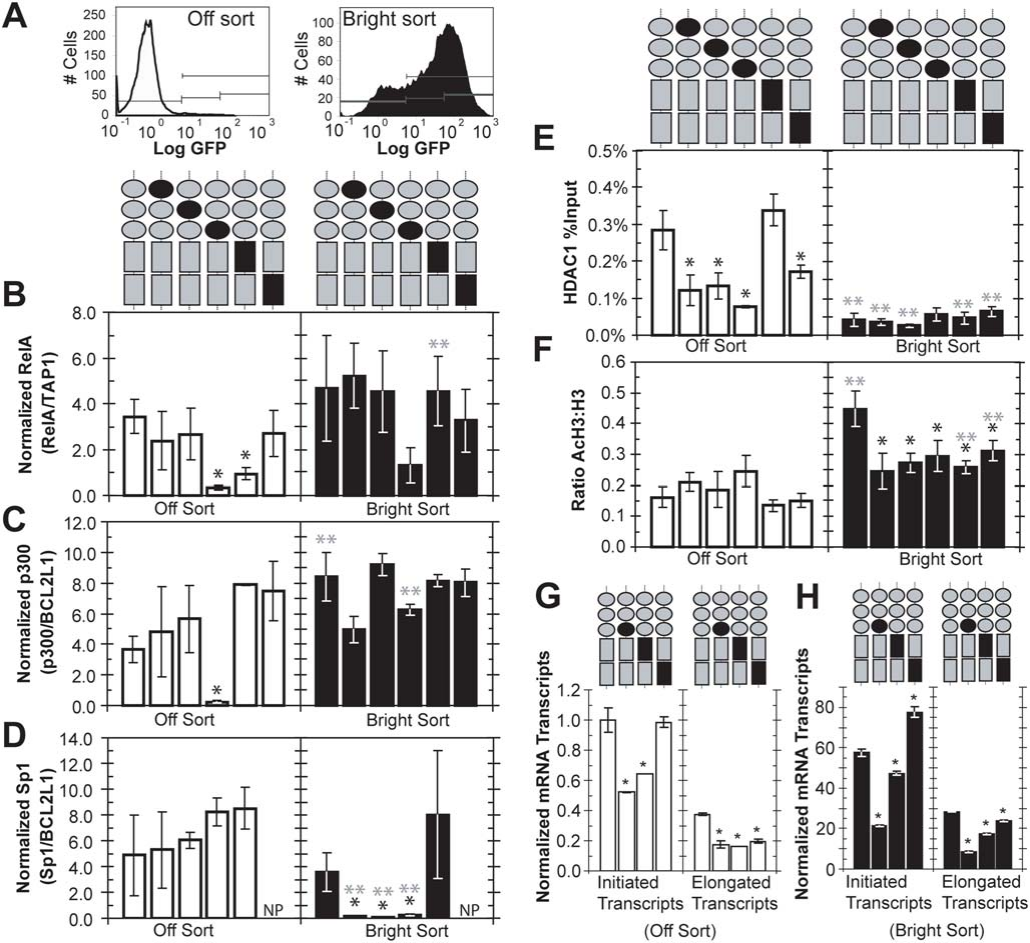
Occupancy of Sp1 and κB Sites in Bright and Off States. (A) Flow cytometry histograms of expanded populations of Off- and Bright-sorted Jurkats infected with *LGIT* and each *LGIT* mutant (as in Figure 3A, panels 5–6). 10^6^ cells were initially sorted from Off and Bright regions, and seven days of expansion was conducted to achieve 5×10^7^ cells necessary for this ChIP protocol. We observed a moderate extent of Bright→Off and Off→Bright dynamic switching over this one-week expansion. (B) RelA ChIP results for Off- and Bright-sorted populations of *LGIT*, *mutI Sp1*, *mutII Sp1*, *mutIII Sp1*, *mutI NF-κB*, and *mutII NF-κB*. Immunoprecipitations were performed using RelA antibody, and immunoprecipitated DNA was quantified using QPCR with primers against the HIV LTR. For analysis of input DNA and RelA immunoprecipitation, all LTR QPCR measurements were normalized by with ChIP-QPCR measurements for the endogenous *TAP1/LMP2* regulatory domain [86], which contains single κB and Sp1 sites that recruit RelA and p50 (refer to Figure S4A and S4B for non-normalized results). Primer sequences and QPCR conditions for HIV LTR and *TAP1/LMP2* are supplied in Materials and Methods and Table S2. The QPCR measurements for LTR and control *TAP1/LMP2* were performed in triplicate, and error bars are standard deviations. Statistically significant differences from WT *LGIT* are denoted by black single asterisks (*, p<0.05), and significant differences between the Off and Bright sorts for any particular mutant is denoted by gray double asterisks (**, p<0.05). (C) p300 ChIP results for Off- and Bright-sorted populations of *LGIT*, *mutI Sp1*, *mutII Sp1*, *mutIII Sp1, mutI NF-κB*, and *mutII NF-κB*. Immunoprecipitations were performed using a p300 antibody, and QPCR measurements were normalized by the endogenous *BCL2L1* regulatory domain [87], which contains Sp1 elements and has been shown to recruit p300 and Sp1 (refer to Figure S4C for non-normalized results). The QPCR measurements for LTR and control *BCL2L1* were performed in triplicate, and error bars are standard deviations. Statistics analyses are the same as in (B). (D) The same experiments as in (C) with a Sp1 antibody. Off- and Bright-sorted populations of *LGIT*, *mutI Sp1*, *mutII Sp1*, *mutIII Sp1*, and *mutI NF-κB* were examined for the presence of Sp1, and QPCR measurements were normalized by the *BCL2L1* regulatory domain (refer to Figure S4D for non-normalized results). *mutII NF-κB* was not performed, as denoted by “NP.” Statistics analyses are the same as in (B). (E) HDAC1 ChIP results for Off- and Bright-sorted populations of *LGIT*, *mutI Sp1*, *mutII Sp1*, *mutIII Sp1*, *mutI NF-κB*, and *mutII NF-κB*. QPCR measurements were normalized by the input DNA. Statistics are the same as in (B). (F) Acetylated histone 3 (lysines 9 and 14) for Off- and Bright-sorted populations of *LGIT*, *mutI Sp1*, *mutII Sp1*, *mutIII Sp1*, *mutI NF-κB*, and *mutII NF-κB*. Total histone 3 (H3) was also quantified by ChIP, and the presented data are the ratios of these QPCR measurements (AcH3/H3). Statistics are the same as in (B). (G) Real time RT-PCR analysis on initiated and fully elongated transcripts for Off-sorted *LGIT*, *mutIII Sp1*, *mutI NF-κB*, and *mutII NF-κB* cell populations. Off sorts were performed as in Figure 6A, and cells were expanded for approximately one week before mRNA extraction. Details for mRNA preparation QPCR are in Materials and Methods and calculations of measurements are discussed in Figure S5. Initiated transcripts were detected with primers for TAR, and elongated transcripts were detected with primers for Tat. Since *mutI Sp1* and *mutII Sp1* did not suggest altered occupancy of p50-RelA heterodimer or p50-p50 homodimer from ChIP experiments, these were not performed. Statistically significant differences from WT *LGIT* are denoted by single asterisks (*, p<0.01). (H) The same experiments as in (G) performed on Bright-sorted *LGIT*, *mutIII Sp1*, *mutI NF-κB*, and *mutII NF-κB*. Bright sorts were performed as in Figure 6A.

ChIP analysis revealed that RelA recruitment to the HIV promoter in Off-sorted cells was reduced approximately 10-fold for *mutIII Sp1* as compared to WT (Figure 6B). In contrast, *mutI Sp1* and *mutII Sp1* promoters recruit RelA to similar extents as WT in both the Off and Bright populations (Figure 6B, p>0.20 compared to respective WT sorts). This finding is consistent with gene expression results suggesting that Sp1 site III is important for recruiting activating complexes, as its mutation led to a higher fraction of Infected but Off cells (Figure 3B), as well as insensitivity to TSA (Figure 5). Therefore, we conclude that Sp1 site III enhances the ability of the κB sites to recruit RelA, and destabilization of the Bright mode in *mutIII Sp1* may in part be due to insufficient recruitment of RelA.

ChIP on *mutI NF-κB* revealed that recruitment of RelA decreased approximately 3-fold for *mutI NF-κB* as compared to WT but was unchanged for *mutII NF-κB* (Figure 6B), confirming distinct roles for the two sites. Also, WT *LGIT*, *mutI NF-κB*, and *mutII NF-κB* recruit RelA to similar extents in the Bright sort (Figure 6B), indicating that κB site I in particular is necessary for the recruitment of p50-RelA heterodimer in the Off mode, but that both κB sites can sufficiently recruit the heterodimer in the Bright mode. ChIP for p50 indicated that WT *LGIT*, *mutI NF-κB*, and *mutII NF-κB* variants recruit p50 to similar extents (Figure S3A), consistent with the fact that p50 is present as part of both the p50-p50 homodimer and the p50-RelA heterodimer (Figure S3B). Thus, ChIP data strongly support the prior hypothesis that κB site I recruits RelA to a greater extent than site II in the Off mode (Figure S3C and S3D). Collectively, our results demonstrate that the two κB sites have distinct roles in transcriptional regulation, and implicate unequal roles in the establishment and maintenance of latency.

### Recruitment of p300 in the Off mode is mediated by Sp1 site III

Histone acetyltransferase p300 is a central factor in HIV transactivation [30] that is actively recruited to the HIV promoter [24] by Sp1 [59] and NF-κB [28],[60] complexes. Analysis of p300 by ChIP revealed similar levels of recruitment for all Bright populations (Figure 6C). However, we observed a ten-fold reduction in p300 recruitment for *mutIII Sp1* relative to *mutI Sp1*, *mutII Sp1*, and WT in the Off-sorted populations, indicating that Sp1 site III is particularly important for recruiting p300 to the HIV promoter in the Off or latent state. Since *mutIII Sp1* suffers a loss of p300 recruitment in the Off mode, the striking insensitivity to TSA stimulation for this mutant (Figure 5) is likely due to the inability to recruit this HAT after inhibition of HDAC activity.

We next analyzed the overall recruitment of Sp1 protein to each LTR in Off and Bright populations. In the Off fraction, the WT promoter and each of the individual Sp1 mutant promoters recruit Sp1 to similar extents (Figure 6D). However, in the Bright fractions, mutation of any of the Sp1 sites results in greater than 10-fold reduction in Sp1 recruitment. This loss of Sp1 in the transactivated (Bright) mode does not correlate with loss of p300 (Figure 6C), suggesting that other factors, including RelA [28],[60] and Tat protein [30],[61], may be involved in the localization of p300.

### Recruitment of HDAC1 in the Off mode is regulated by Sp1 site III

Transcriptional repression is commonly regulated by histone deacetylation, and HDAC1 is associated with p50-p50 homodimer [3] and Sp1 [39] at the HIV-1 LTR. Therefore, we performed ChIP against HDAC1 to determine its recruitment to each Sp1 and κB site and its role in transcriptional repression (Off sorts) vs. activation (Bright sorts). ChIP on the Off sorts revealed statistical decreases in HDAC1 occupancy for all mutants, except *mutI NF-κB*, when compared to WT *LGIT* (Figure 6E). In contrast, HDAC1 occupancy in the Bright sorts was statistically indistinguishable from WT for all mutants (p>0.1, Figure 6E). Additionally, WT and all mutants except *mutIII Sp1* had elevated levels of HDAC1 occupancy in the Off sorts compared to the respective Bright sorts (p<0.1, Figure 6E). Collectively, these ChIP findings reveal that each Sp1 site and κB site II are important in the recruitment of HDAC1, and that mutation of Sp1 site III abolishes differential regulation of HDAC1 between Off and Bright modes.

### All Sp1 and κB elements are required for maximum acetylation of H3

To estimate differences in overall levels of transcriptional activation between Off and Bright sorts and between WT and mutant populations, we measured acetylation of the histone 3 tail at lysines 9 and 14 (AcH3) by ChIP and normalized to total histone 3 (Figure 6F). In the Off sort, each mutant was statistically indistinguishable to WT *LGIT* (p>0.05, Figure 6F). In contrast, the Bright sorts revealed significant decreases of AcH3 for all mutants compared to WT. These results show that each Sp1 and κB site is essential for maximum acetylation of H3. However, there are no significant differences in AcH3 between Off and Bright sorts of *mutI Sp1*, *mutII Sp1*, and *mutIII Sp1* cells, indicating that each of the three Sp1 sites is required for the regulation of the deacetylated (Off) and acetylated (Bright) states.

Lastly, to examine an indicator of repressed chromatin beyond histone deacetylation, we performed ChIP for Off and Bright sorts to examine trimethylation of histone 3 at lysine 9 (TriMetH3K9). However, we observed undetectable levels of TriMetH3 for the Off and Bright sorts of WT *LGIT* and all Sp1 and κB mutants (Figure S4E), suggesting that trimethylation of H3K9 is not a significant factor in the phenotypes we observe in *LGIT*, including dynamic switching and stabilization of the Off mode.

### Reduction of RelA recruitment decreases transcriptional initiation and elongation

Sp1 site III regulates recruitment of repressor HDAC1 (Figure 6E) and activators p300 (Figure 6C) and p50-RelA (Figure 6B). In contrast, RelA occupancy is not hindered by mutation of κB site II; however, mutation of κB site I decreases the occupancy of p50-RelA heterodimer but apparently not p50-p50 homodimer (Figure 6B and Figure S3C and S3D). The differences in p50-p50, p50-RelA, p300, and HDAC1 occupancies at these mutant promoters likely influence the frequencies of both transcription initiation and elongation at the LTR. In particular, an inactive LTR occupied with a repressive p50-p50 homodimer and HDAC1 would be unable to recruit RNAPII and would thus not initiate transcription [3]. In contrast, when p50-RelA heterodimer and p300 localize to the LTR, RNAPII is readily recruited and phosphorylated by P-TEFb, to generate fully elongated, productive transcripts [3],[19]. Competition between the recruitment of repressing p50-RelA heterodimer and p50-p50 homodimer may result in RNAPII initiating transcription and then stalling, which results in a large number of abortive transcripts and little *GFP* and *Tat* expression [3],[19]. Thus, we adapted a previously established RT-PCR method [3] to quantify the functional differences transcriptional initiation and elongation for the Off and Bright sorts of WT *LGIT*, *mutIII Sp1*, *mutI NF-κB* and *mutII NF-κB*.

The Off sorts for *mutI NF-κB* and *mutIII Sp1* have respective decreases of 36% and 48% in the number of initiated transcripts compared to WT (Figure 6G), in agreement with ChIP observations that these two mutants have decreased p50-RelA occupancy and potentially increased recruitment of the repressing p50-p50 homodimer (Figures 6B and S3C). In contrast, there is no statistical change in transcriptional initiation for the *mutII NF-κB* Off sort (p = 0.88 vs. WT, Figure 6G), also in agreement with ChIP observations of no change in p50-RelA occupancy vs. WT (Figure 6B). Quantification of transcriptional initiation on Bright-sorted populations revealed a similar trend, in which *mutII NF-κB* exhibited a 35% increase in the number of initiated transcripts compared to WT, while *mutI NF-κB* and *mutIII Sp1* have significant decreases (18% and 63%, respectively, Figure 6F).

Although transcription initiation was not hindered for *mutII NF-κB* (and was actually enhanced in the Bright mode), this mutant has a 48% decrease in the number of elongated transcripts vs. WT in the Off sort and a 15% decrease in the Bright sort (Figure 6E and 6F). Mutation of κB site I further decreased transcriptional elongation, with 57% and 38% decreases for Off and Bright sorts vs. WT, respectively (Figure 6E and 6F). These findings demonstrate that while both κB sites are required for full transcriptional elongation, κB site I has a greater contribution in both transcriptional initiation and elongation. Importantly, mutation of Sp1 site III has striking reduction of transcriptional elongation in the Off and Bright modes (53% and 70% decreases, respectively, Figure 6E and 6F), consistent with ChIP observations that Sp1 site III is necessary for recruitment of RelA in both Off and Bright modes and of p300 in the Off mode (Figure 6B). Moreover, these data indicate that κB site I and Sp1 site III combine to activate transcriptional initiation (likely in part via recruitment of p50-RelA heterodimer), and mutation of either site abrogates this role.

### Summary of molecular and transcriptional phenotypes

**Figure 7:**
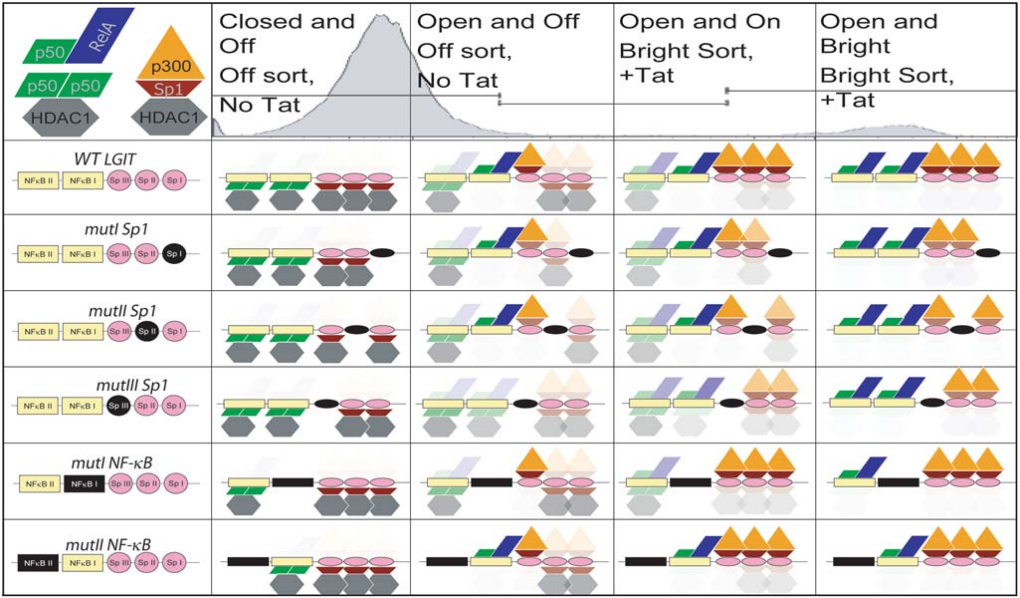
Model of Sp1 and κB Occupancy in Off, Bright, and Intermediate Regions. This cartoon model proposes the localization of chromatin factors to the Sp1 and κB sites within the HIV-1 LTR. The NF-κB dimers (p50-p50 or p50-RelA) lead to differential recruitment of HDAC1 or p300, respectively. Likewise, the Sp1 protein has been demonstrated to recruit either activating HATs (such as p300) or repressing HDACs (such as HDAC1). The structural conformation and association with either HDAC1 or p300 may govern the DNA-binding affinity of Sp1, and we have illustrated these two conformations by the orientation of the Sp1 molecule (maroon trapezoid). Other symbols include p300 (orange triangle), RelA (blue parallelogram), p50 (green rhombus), and HDAC1 (gray hexagon). The localization of repressing factors (illustrated below Sp1 and κB elements) is enhanced in the Off mode and the presences of activating factors (above Sp1 and κB elements) is enhanced in the Bright mode. Note that κB site I and Sp1 site III recruit RelA and p300, respectively, and these two sites appear to have an important synergistic and/or cooperative role in transcriptional activation.

Collectively, the molecular and transcriptional phenotypes reveal that mutation of any Sp1 site destabilizes the Off and Bright gene expression modes and increases dynamic switching and phenotypic bifurcation. Alternatively, mutation of κB site I appears to slightly increase the stability of the Off mode, while mutation of κB site II may slightly stabilize the Bright mode. The molecular regulation of Off and Bright modes of each *LGIT* variant is summarized in Figure 7, which integrates the results from ChIP, perturbation, and transcriptional experiments into a model of transcription factor occupancies.

In Figure 7, the degree of shading (or transparency) of the individual molecules corresponds to hypothetical degrees of occupancy. For this study, these configurations are inferred by functional tests (transcriptional activities and responses to perturbation) and directly measured by ChIP, which are all summarized in Figure S6. We hypothesize that repressing markers, including p50-p50 homodimer, HDAC1, and deacetylation of lysines 9 and 14 of histone 3 (H3K9/14), indicate a stabilized Off mode and inactive transcription. In contrast, we hypothesize that a stabilized Bright mode and transcriptional activation are characterized by the presence of p50-RelA, p300, and histone acetylation of H3K9/14. However, the association of Sp1 with HDAC1 or p300 may govern its structural conformation and DNA-binding affinity [35],[55],[62], and we have illustrated these two conformations by the orientation of the Sp1 molecule (trapezoid). The strongest destabilizing phenotypes occur with *mutIII Sp1*, which displays the highest population of Infected but Off cells, highest Mid:On ratio, and lowest Bright Mean position; exhibits the strongest response to TNF-α but the weakest to TSA; yields the highest frequency of phenotypic bifurcation and greatest dynamic switching; and recruits decreased levels of RelA (Off mode), p300 (Off mode), HDAC1 (Off mode) and Sp1 (Bright mode) (Figures 7 and S6).

## Discussion

We have previously demonstrated that stochastic fluctuations in Tat levels may contribute to HIV-1 proviral latency by delaying the onset of viral gene expression and Tat feedback [9]. In this study, we explored how each Sp1 and κB element in the HIV LTR modulates these stochastic fluctuations via differential recruitment of activating and repressing factors. We found that each Sp1 and κB site contributes distinctly to the dynamics of switching between low and high gene expression modes and the frequency of phenotypic bifurcation—both of which have implications for viral latency.

This experimental system, based on single integrations of the *LGIT* provirus in CD4^+^ Jurkat cells [9], is similar in design to the J-Lat model—a clonal Jurkat cell line with a single integration of full-length HIV in which the viral gene *Nef* has been replaced by *GFP*—which has been used as an *in vitro* model for HIV latency [3],[32],[46],[63]. In this study, individual Sp1 and κB elements within the LTR of *LGIT* were disabled to dissect their contributions to transcriptional activation and repression. Furthermore, this system examines the dynamic behavior of single integrated proviruses rather than transient transfection reporter systems [40]–[42]—which provide valuable gene expression information but do not account for the integration site, chromatin environment, and low copy number of the provirus—or viral replication assays [43]–[45]—which measure replicative fitness but not gene expression or proviral latency. Additionally, by isolating and characterizing transactivated (Bright) and latent (Off) populations, we examine the contribution of each Sp1 and κB site to transactivated and “latent” states, as well as their responses to stimulation with pharmacological agents TNF-α and TSA.

Gene expression results (Figures 2–4) provide corroborating evidence that mutation of *any* one of the three Sp1 binding sites dramatically increases promoter sensitivity to transcriptional noise and stochastic effects in the Tat feedback loop. Moreover, we conclude that each Sp1 site—particularly Sp1 site III—plays an important role in the control of stochastic gene expression by stabilizing the inactive and active transcription states via the recruitment of activating and repressing factors to the LTR (Figure 6). In support of a destabilized Bright state, single Sp1 mutations and in particular site III mutation result in a significantly weaker promoter (Figure 2A), a 2- to 3-fold increase in the Off (latent) population of infected cells (Figure 3B), rapid switching from Bright to Off (Figure 3C), and a considerable loss of overall Sp1 binding to the promoter in the Bright state as measured by ChIP (Figure 6D). As support for a destabilized Off state, Sp1 mutations result in rapid switching from Off to Bright (Figure 3D), a considerable increase in the fraction of integrated provirus that responds to TNF-α (Figure 5), and a decrease in the LTR occupancy of HDAC1 as measured by ChIP (Figure 6E). In addition, as evidence of both destabilized Off and Bright modes for the Sp1 mutants, there were insignificant differences between the deacetylated vs. acetylated states of the respective Off and Bright sorts (Figure 6E). Collectively, these destabilizing effects result in a promoter more susceptible to transcriptional noise and phenotypic bifurcation (Figure 4A), with potentially important implications for viral latency.

We also demonstrate that the two κB sites, despite having identical sequences, provide distinct roles in transcriptional activation. κB site I plays a preferential role in promoter activation, likely because cooperativity between κB site I and the adjacent Sp1 site III promotes recruitment of p50-RelA and p300 (Figure 6B and 6C). In contrast, the distal κB site II provides a bias for the recruitment of p50-p50 homodimer (Figure S3C) and HDAC1 (Figure 6E). These observations bear similarities to TLR-induced genes in dendritic cells, in which subunit specificities of κB sites are governed by cooperative interactions with other factors, including CBP [64].

Binding cooperativity between Sp1 and RelA has been reported in biochemical studies [65],[66], and the distinct roles we have observed for the different Sp1 and κB binding sites raises the possibility that the individual enhancer sites may differentially contribute to this cooperativity. ChIP results show that p300 recruitment to the *mutI NF-κB* promoter is maintained, whereas RelA is lost; however, both p300 and RelA are lost in *mutIII Sp1* (Figure 6B and 6C). This result implies that Sp1 site III may first recruit p300, whose presence enhances p50-RelA localization to κB site I. The proximity of κB site I to Sp1 site III may even underlie its biased recruitment of the p50-RelA heterodimer, which may be related to reported direct binding between RelA and Sp1 [67] or between RelA and p300 [28],[29].

Although Sp1 site III is required for p300 recruitment in the Off mode, other factors at the LTR may contribute to subsequent p300 maintenance at the promoter [35]. These may include p300 interacting with RelA [68], LEF-1 [38], YY1 [69], and SWI/SNF [25],[60], as well as p300 directly binding to DNA at motifs (i.e., GGGANT) found within the LTR, including in both κB elements [70]. RelA and p300 are bound to the LTR in all Bright sorts, and also in most Off sorts, supporting earlier models that RelA and p300 are necessary, but not sufficient, for Tat-transactivation, and recruitment of other factors (RNAP II, P-TEFb, PCAF, etc.) is required [31],[71]. Other potential factors contributing to RelA localization at the promoter in the Off mode include its binding to an inactive promoter [72], its potential interaction with various HDACs [71],[73],[74], or the competing repressive roles of other regulatory factors (YY1, LSF/LBP-1, etc.) [75]–[77].

Sp1 recruitment to WT *LGIT* was unchanged in Off vs. Bright cells, as assessed by ChIP (Figure 6D). This finding corroborates a recent study in transiently transfected cells that detected no change in Sp1 levels at the LTR with or without addition of exogenous Tat [58]. In contrast to the WT LTR, Sp1 recruitment to the Sp1 mutant promoters was compromised in the Bright sorts relative to the Off sorts, potentially due to different Sp1 binding affinities in the two expression modes. In the Bright sorts, the association of individual Sp1 molecules with p300 may weaken its binding affinity for DNA [35],[62]. In contrast, Sp1 interactions with HDAC1 appear to have no reduction in DNA binding [55]. The DNA binding affinities of Sp1 are increased by homomultimerization and synergy between Sp1 molecules [67],[78], and Sp1 EMSA analyses with the HIV-1 LTR have revealed that mutation of one of the three Sp1 sites reduced Sp1 recruitment, while mutation of two sites eliminated detection of Sp1 [79]. Thus, we hypothesize that the diminished Sp1 levels in Bright sorts of Sp1 mutants may result from decreased affinities of individual Sp1 molecules and failure to recruit multimerized Sp1 complexes.

Collectively, these findings demonstrate that the balance between the repressing and activating roles of each Sp1 site impacts transcriptional noise and the propensity for latent infections. Therefore, it appears that by recruiting activating and repressing host factors, intact Sp1 sites dampen noise in HIV gene expression. Although it remains to be determined whether such noise arises from events external to (extrinsic) or directly from (intrinsic) the mechanisms under study here [80], the Sp1 sites' regulation of promoter activity and chromatin dynamics agrees with a paradigm that eukaryotic promoters generate noise by localization of chromatin factors [12],[13]. Such local chromatin dynamics may yield transcriptional pulses [16],[17] that would be amplified by Tat feedback.

Although the Sp1 and κB sites enhance replicative fitness of the virus [43]–[45],[50], variations and mutations within these sites are often observed in isolates from subtype B cohorts [79],[81],[82]. Moreover, there is considerable sequence variability of Sp1 and κB elements across different HIV-1 subtypes [82],[83]. In addition to altering the replication fitness of the virus, our findings suggest that such evolutionary divergence within subtype B variants and across other subtypes likely impact viral transcriptional dynamics and propensities for latency. Thus, we postulate that variations in promoter architectures will have important unexplored epidemiological and therapeutic implications.

This work demonstrates the power of quantitative, dynamic phenotyping of viral mutants for dissecting regulatory inputs into the viral promoter in a proviral model of HIV. This approach revealed that each Sp1 site influences the control of stochastic gene expression by stabilizing both the active state—therefore likely playing a role in the regulation of bursts in viral gene expression—and the inactive state—thus playing a role in the establishment and maintenance of proviral latency. It remains to be determined which of these features are central to survival and propagation of the virus in a natural environment or under therapeutic challenge. Finally, this work may aid the future development of paradigms to predict the gene expression and latency phenotypes of HIV-1 isolates and subtypes, as well as draw important correlations between viral genotype and clinical outcomes and responses to antiviral therapies.

## Materials and Methods

### Plasmids

Construction of *LGIT* plasmids has been previously described [9]. Double and triple point mutations at the Sp1 and κB sites in the HIV LTR were performed using the Quikchange PCR method (Stratagene). The specific inactivating mutations used for κB [50] and Sp1 [43],[48],[49] were previously described, and primer sequences are listed in Table S1.

### Cell culture and chemical perturbations

Jurkat cells were cultured in RPMI 1640 (Mediatech) medium supplemented with 10% fetal bovine serum, 100 U/ml penicillin-streptomycin, and 2 mM L-glutamine. Cells were grown at concentrations between 2×10^5^ and 10^6^ cells/ml in 5% CO_2_ at 37^o^C. HEK 293T cells, used for lentiviral packaging, were cultured in the same conditions as Jurkats but with Isocove's DMEM (Mediatech). For perturbation and viral titering experiments, the following factors were used in the specified concentrations: 20 ng/ml tumor necrosis factor-α (TNF-α, Sigma-Aldrich), 400 nM trichostatin A (TSA, Sigma-Aldrich), and 5 mM hexamethylene bisacetamide (HMBA, Sigma-Aldrich).

### Viral harvesting and infection

Lentiviral vectors were packaged and harvested in HEK 293T cells using 10 µg of pCLGIT (or mutant κB/Sp1 variants), 5 µg pMDLg/pRRE, 3.5 µg pVSV-G, and 1.5 µg pRSV-Rev, as previously described [9],[84], then concentrated by ultracentrifugation to yield between 10^7^ and 10^8^ infectious units/ml. For titering, 3×10^5^ Jurkat cells in 12-well plates were infected with approximately 10^3^–10^6^ infectious units per well. Six days later, infected Jurkats were incubated with a combination of 5 mM HMBA, 20 ng/ml TNF-α, and 400 nM TSA for 18 hours and then analyzed by flow cytometry to determine infectious titer by GFP expression. This combination of agents was chosen to stimulate the promoter via P-TEFb [85], NF-κB [36], and Sp1 dependent mechanisms [37]. Titering curves were constructed to achieve infection of 5–10% of cells after maximum stimulation, corresponding to MOI ∼0.05–0.10.

### FACS analysis and sorting

Infected cultures were analyzed via flow cytometry on a Beckman-Coulter EPICS XL-MCL cytometer (http://biology.berkeley.edu/crl/cell_sorters_analysers.html). All flow measurements were performed in parallel with an uninfected Jurkat control, and perturbation experiments with TNF-α and TSA were performed in parallel with stimulated but uninfected Jurkat controls. To isolate infected, expressing populations, GFP^+^ cells were sorted on a DAKO-Cytomation MoFlo Sorter. As described in the text, bulk population (polyclonal) and single cell (clonal) sorts were performed for a range of different GFP positive regions, as follows: *LGIT* bulk and clonal sorts: “Off” region (∼0.1–2.0 Relative Fluorescence Units), “Mid” region (∼2.0–30 RFU), “Bright” region (∼30–1024 RFU), and “On” region (∼2.0–1024 RFU). Flow cytometry data analysis was performed with FlowJo (Tree Star, Inc.).

### Analysis of gene expression by flow cytometry

Gene expression levels were tracked over a 21-day time course by measuring the fluorescence intensity of GFP in *LGIT* and mutant cells. Cells were infected at an MOI ∼0.05–0.10, and a GFP+ population was detectable by flow cytometry 48 hours after infection. The time to peak activity occurred approximately one week after infection; however, a bimodal distribution of infected cells (“Off” and “Bright”) persisted throughout the three week experiment. The strength of Tat-transactivation was measured by examining the Bright population, and in particular, the mean of this population (Bright Mean) was used as a marker of the base efficiency of transactivated gene expression. The Bright Mean of infected *LGIT* and mutants was determined by calculating the average relative fluorescence of cells within the “Bright” region (∼30–1024 RFU).

For *LGIT* and all mutants, a small fraction of cells occurs in a critical region defined as the Mid region (∼2.0–30 RFU), which lies between Off (∼0.1–2.0 RFU) and Bright (∼30–1024 RFU) populations. Cells isolated from this region tend to turn Off or Bright in a random fashion, demonstrating an instability of that region [9]. The fraction of infected cells persisting in the Mid region at a specific time is represented by the Mid:On ratio, in which the On region (∼2.0–1024 RFU) is the sum of Mid and Bright subpopulations. We employ this ratio as a metric for transcriptional instability, such that a high Mid:On ratio suggests an unstable promoter and a high degree of stochastic switching.

### Chromatin immunoprecipitation and quantification by QPCR analysis

FACS sorting was performed to isolate Off and Bright fractions of the *LGIT* and *LGIT* mutant cell lines, and 1×10^6^ cells were acquired for each sort. Off, Bright, and original unsorted populations were expanded to achieve 5×10^7^ cells, incubated in 1% formaldehyde for 10 minutes at room temperature for fixation, and subsequently incubated with 125 mM glycine for 5 minutes at room temperature to quench the formaldehyde. Upstate EZ ChIP (17–371) reagents and protocol were utilized for crosslinking, lysis, sonication, immunoprecipitation, elution, reverse crosslinking, and DNA purification procedures. Sonication was performed with the Branson Sonifier 450 for 15 cycles with power output of 2.5, 10% duty cycle, for 10–15 second pulses and 1 minute intervals on ice. DNA was sheared to achieve an average of 0.2–0.7 kb, as confirmed by DNA gel electrophoresis. Immunoprecipitations were performed with Upstate polyclonal antibodies anti-p50 (06–886), anti-p65 (06–418), anti-p300 (05–257), anti-Sp1 (07–645), and anti-AcH3H9/14 (06–599) and Abcam polyclonal antibodies anti-HDAC1 (ab7028), anti-H3 (ab1791), and anti-TriMetH3K9 (ab8898).

Immunoprecipitated DNA was quantified using quantitative polymerase chain reaction (QPCR) with primers within the HIV LTR which flank the κB and Sp1 elements [46]. To accurately assess the input of each QPCR reaction, and to normalize for the efficiency of immunoprecipitation of each antibody, we used endogenous promoters containing functional κB and/or Sp1 domains as normalization controls for RelA, p300, and Sp1. The endogenous *TAP1/LMP2* regulatory domain (PubMed accession# NM_000593.5), which contains a single κB site four nucleotides downstream of an Sp1 site, was used to normalize QPCR data from RelA and p50 immunoprecipitations, as this promoter has been shown to constitutively recruit both p50-RelA and Sp1 [86]. Similarly, the endogenous *BCL2L1* regulatory domain (PubMed accession# NW_001838664.2), which contains Sp1 elements and has been shown to strongly recruit p300 and Sp1 [87], was used for normalizing QPCR data from p300 and Sp1 immunoprecipitations. Non-normalized ChIP results are presented in Figure S4, and all ChIP primer sequences are provided in Table S2. HDAC1, AcH3, TriMeH3, and H3 immunoprecipitations were normalized by inputs, and acetylated histone 3 is reported as a ratio of AcH3:H3 immunoprecipitations.

Amplified DNA products from each primer set were cloned into the Invitrogen pCR2.1 plasmid (pCR2.1-TOPO-LTRκB, pCR2.1-TOPO-TAP1/LMP2, and pCR2.1-TOPO-BCL2L1) to create plasmids that were subsequently used to generate standard curves for all QPCR analyses. Linear regression of standard curves was achieved by serial dilutions ranging from ∼10 ng to ∼10^−6^ ng plasmid DNA, which corresponds to ∼2×10^9^ to ∼2×10^2^ copies per 20 µL reaction. Quantitative PCR was performed using the iCycler iQ Real-Time PCR Detection System (Bio-Rad, Hercules, CA), and SYBR Green I (Invitrogen) was used as the fluorescent nucleic acid stain.

### mRNA extraction and quantification by RT-PCR

As performed in ChIP experiments, FACS sorting was used to isolate Off and Bright fractions of the *LGIT* and *LGIT* mutant cell lines, and 1×10^6^ cells were acquired for each sort. Off and Bright populations were expanded to achieve 1×10^7^ cells, total mRNA was isolated using Trizol (Invitrogen), and transcripts were quantified using the QuantiTect SYBR Green RT-PCR kit (Qiagen) on the Bio-Rad iCycler. The total number of transcripts (initiated and elongated) were detected with TAR primers [3], and *Tat* primers were used to detect only elongated transcripts (sequences in Table S3). For each sample, initiated and elongated transcript levels were normalized by the corresponding levels of β-Actin mRNA (sequences in Table S3) [9]. Measurements and calculations of initiated, elongated, and truncated transcripts are provided in Figure S5. Triplicate RT-PCR measurements were performed for all samples for each primer set, and melt curves were performed on the Bio-Rad iCycler for all samples to confirm the specificity of QPCR reaction.

## Supporting Information

Figure S1 Transcriptional Profiles of *LGIT* and *LGIT* Mutant Time Course Infections. (A) As described in Figure 2, *LGIT* and corresponding Sp1 and κB mutants were infected in Jurkat cells at low MOI (∼0.05–0.10) in biological triplicate, and GFP expression was monitored over a 21-day time course. Histograms from each replicate for each day were used to generate a heat map for days 2–13 of the time course. The heat map indicates the distribution of GFP fluorescence (y-axis) for each *LGIT* variant and how this distribution changes over time (x-axis). The heat map reflects the GFP fluorescence beyond the autofluorescence threshold, which is set at 2.0 relative fluorescence units (RFU). The depicted region is the sum of “Mid” and “Bright” regions (Figure 1C), and is termed the “On” region. Further details of data analyses are in available in Materials and Methods. Two *LGIT* variants (*mutALL Sp1* and *mutIII Sp1/mutI NF-κB*) failed to generate a GFP+ population of cells after infection at low MOI (∼0.05–0.10) and were thus omitted from this study (see Figure S1B). (B) *LGIT* and mutant infections (MOI ∼0.05–0.15) are shown seven days after infection (filled grey). In parallel, each population was stimulated with TNF-α (red outline) or TSA (blue outline) for 18 hours six days after infection. In addition to the previously mentioned Sp1 and κB *LGIT* mutants, two other mutant combinations (mutation of all Sp1 sites, *mutALL Sp1*, and mutation of Sp1 site III and κB site I, *mutIII Sp1/mutI NF-κB*) failed to generate a Bright population after infection and were negligibly responsive to stimulation with TNF-α or TSA. Since infections with these particular *LGIT* mutants failed to generate a Bright mode, they were omitted from further study, as the focus of this investigation was the molecular and functional differences between Off and Bright modes in a bimodal population.
(12 MB TIF)

Figure S2 Increased Switching Dynamics for Sp1 Mutant PheB Clones. (A) Transcriptional switching dynamics were measured for one PheB clone for each LGIT variant. Off (red outline) and Bright (blue outline) fractions were isolated with FACS, and flow cytometry was used four days after sorting to measure the degree of switching (see Figure 4D). Off and Bright switching measurements were normalized by the distribution of cells in the unsorted PheB clone (gray filled histogram). Cytometry measurements for the unsorted PheB clones were performed at the same time as the sorts. (B) Same as (A) with flow cytometry measurements performed seven days after sorting. Cytometry measurements for the unsorted PheB clones were performed at the same time as the sorts. Refer to Figure 4E for quantitative results.
(2.1 MB TIF)

Figure S3 Latent *mutI NF-κB* Retains Occupancy of p50-p50 Homodimer but not p50-RelA Heterodimer. (A) p50 ChIP results for Off-sorted and Bright-sorted populations of *LGIT*, *mutI NF-κB*, and *mutII NF-κB* as well as TNF-α-stimulated WT *LGIT*. Immunoprecipitations were performed using p50 antibody, and immunoprecipitated DNA was quantified using QPCR with primers against the HIV LTR. For normalization of input DNA and p50 immunoprecipitation, all LTR QPCR measurements were normalized by with ChIP-QPCR measurements for the endogenous *TAP1/LMP2* regulatory domain, which contains single κB and Sp1 sites recruits RelA and p50 (20). Primer sequences and QPCR conditions for HIV LTR and *TAP1/LBP2* are supplied in Materials and Methods. The QPCR measurements for LTR and control *TAP1/LBP2* were performed in triplicate, and error bars are standard deviations. For all panels in Figure S3, statistically significant differences from the corresponding sort for WT *LGIT* are denoted by single asterisks (*, p<0.01) and double asterisks (**, p<0.05). (B–D) Calculations of p50-p50 homodimers and p50-RelA heterodimers for Off-sorted and Bright-sorted populations of *LGIT*, *mutI NF-κB* and *mutII NF-κB* and TNF-α-stimulated WT *LGIT*. WT *LGIT* was stimulated with TNF-α (20 ng/ml, incubated for 30 minutes before crosslinking), and ChIP was performed using p50 and RelA antibodies. Since TNF-α induces nuclear localization of RelA, for the purposes of an approximate calculation, all NF-κB bound to the κB sites of the LTR can be approximated as p50-RelA heterodimer and none as p50-p50 homodimer. With this assumption, and the supposition that stimulation with TNF-α results in the maximum induced levels of p50 and RelA bound to the HIV LTR, we calculated the maximum normalized concentrations of p50 (p50_max_ = 1.86) and RelA (RelA_max_ = 7.6). The ratio of induced measurements of RelA to p50 (7.6/1.86 = 4.1) corresponds to the relative efficiency in RelA and p50 immunoprecipitations. Let the variables *x* and *y* be the concentrations of p50-p50 homodimer and p50-RelA heterodimer, respectively. Let *z* be the total concentration of NF-κB, so that *z* = *x*+*y*. Since RelA only exists in the p50-RelA heterodimer form, *y* is equal to be the normalized measurement of RelA from ChIP. Since both p50-p50 homodimer and p50-RelA heterodimer contain p50, then *z* = (RelA_max_/p50_max_)×p50_sample_, or *z* = 4.1×p50_sample_. Thus, *x* = *z*–*y*, so *x = *4.1×p50_sample_–RelA_sample_. Calculations of total NF-κB (*z*, panel B), p50-p50 homodimer (*x*, panel C), and p50-RelA heterodimer (*y*, panel D) were performed for Off-sorted and Bright-sorted populations of *LGIT*, *mutI NF-κB*, and *mutII NF-κB*.
(1.5 MB TIF)

Figure S4 Non-normalized ChIP QPCR as a Percentage of Input DNA. (A) As performed in Figure S3, ChIP QPCR results as a percentage of input against p50 for Off- and Bright-sorted *LGIT*, *mutI NF-κB*, and *mutII NF-κB* and TNF-α-induced WT *LGIT*. Denoted are the HIV LTR (white bars) and the control gene, *TAP1/LBP2* (gray bars). *mutI Sp1*, *mutII Sp1*, and *mutIII Sp1* were not performed. (B) As performed in Figure 6B, ChIP QPCR results as a percentage of input DNA against RelA for Off- and Bright-sorted *LGIT*, *mutI Sp1*, *mutII Sp1*, *mutIII Sp1*, *mutI NF-κB*, and *mutII NF-κB* and TNF-α-induced WT *LGIT*. Denoted are the HIV LTR (white bars) and the control gene, *TAP1/LMP2* (gray bars). This is the same control gene as used for p50. (C) As performed in Figure 6C, ChIP QPCR results as a percentage of input against p300 for Off- and Bright-sorted *LGIT*, *mutI Sp1*, *mutII Sp1*, *mutIII Sp1*, *mutI NF-κB*, and *mutII NF-κB*. Denoted are the HIV LTR (white bars) and the control gene, *BCL2L1* (gray bars). (D) As performed in Figure 6D, ChIP QPCR results as a percentage of input against Sp1 for Off- and Bright-sorted *LGIT*, *mutI Sp1*, *mutII Sp1*, *mutIII Sp1*, and *mutI NF-κB*. Denoted are the HIV LTR (white bars) and the control gene, *BCL2L1* (gray bars). This is the same control gene as used for p300 and *mutII NF-κB*, denoted as “NP”, was not performed. (E) ChIP QPCR results as a percentage of input against trimethylated histone 3 lysine 9 (TriMetH3K9) for Off- and Bright-sorted *LGIT*, *mutI Sp1*, *mutII Sp1*, *mutIII Sp1*, *mutI NF-κB*, and *mutII NF-κB*. Off-sorts are denoted by white bars, Bright-sorts are denoted by black bars, and uninfected Jurkat is denoted by the gray bar. Note that no samples exceed the background (uninfected) control, indicating that TriMetH3K9 is below detection levels.
(1.8 MB TIF)

Figure S5 Calculations of Truncated Transcripts by RT-PCR. (A–B) As performed in Figures 6G–H, real time RT-PCR analysis on initiated and fully elongated transcripts for Off- and Bright-sorted *LGIT*, *mutIII Sp1*, *mutI NF-κB*, *mutII NF-κB*, and *mutI&II NF-κB*. Off and Bright sorts were performed as in Figure 6A, and cells were expanded for approximately one week before mRNA extraction. Details for mRNA preparation QPCR are in Materials and Methods in the main text. Initiated transcripts were detected with primers for TAR, and elongated transcripts were detected with primers for Tat. For both panels, statistically significant differences from the corresponding sort for WT *LGIT* are denoted by single asterisks (*, p<0.01) and double asterisks (**, p<0.05).
(0.92 MB TIF)

Figure S6 Stabilizing and Destabilizing Phenotypes for Off and Bright Modes. (A) Summary of molecular and transcriptional phenotypes in the inactive (Off) transcriptional mode. Measurements for WT *LGIT* are assigned a “0” value, and comparisons for each mutant to WT *LGIT* are denoted by increases (+), decreases (−), and unchanged (0) values. Potential changes to the stabilities of the Off mode are indicated by green (stabilizing) and red (destabilizing) boxes. Measurements denoted with “NP” (black boxes) were not performed. (B) Same as in (A) for the active (Bright) transcriptional mode. Potential changes to the stabilities of the Bright mode are indicated by green (stabilizing) and red (destabilizing) boxes. Measurements denoted with “NP” (black boxes) were not performed. (C) Differential regulation of the Off and Bright modes was determined by examining phenotypic differences in the Off- and Bright-sorts and dynamic switching between modes. For ChIP measurements, statistically significant increases (+), decreases (−), and unchanged (0) values for Bright sorts, compared to the Off-sorted fractions, are denoted. For switching and dynamics phenotypes, mutant values are compared to the WT *LGIT* counterpart, as in (A) and (B). Differential regulation of Off and Bright modes is indicated by green (strongly regulated) and red (disregulated) boxes. Measurements denoted with “NP” (black boxes) were not performed.
(1.5 MB TIF)

Table S1 Quikchange Primer Sequences for *LGIT* mutants
(0.04 MB DOC)

Table S2 Primer Sequences for ChIP QPCR
(0.03 MB DOC)

Table S3 Primer Sequences for Quantitect RT-PCR
(0.03 MB DOC)

Table S4 Non-normalized Bright Mean Position (RFU)
(0.03 MB DOC)
