# Supplementary figures and images for: Control of Stochastic Gene Expression by Host Factors at the HIV Promoter
Source: PLoS Pathog. 2009 Jan 9;5(1):e1000260. doi: 10.1371/journal.ppat.1000260 (PMC2607019; doi:10.1371/journal.ppat.1000260)

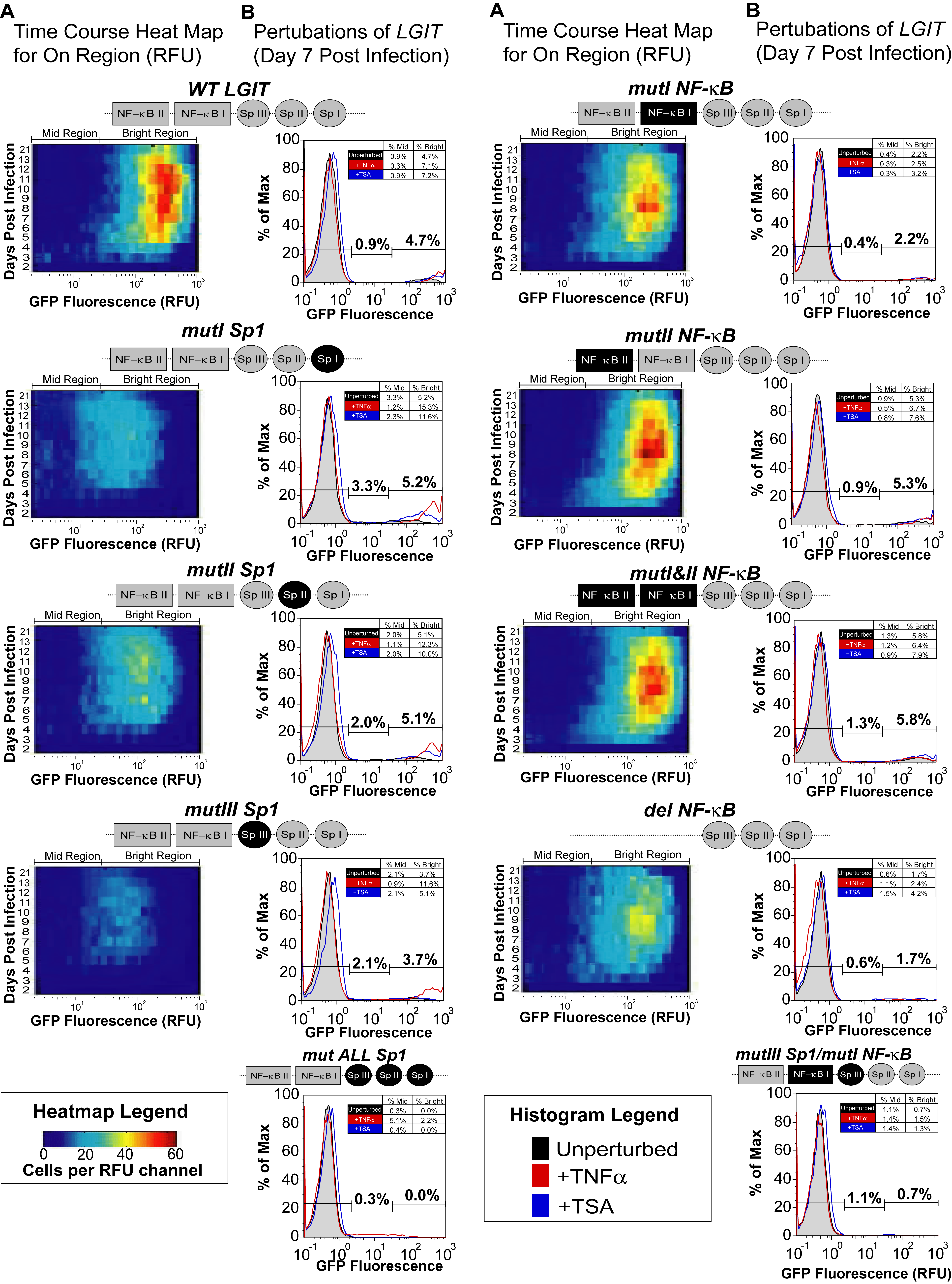

Supplement: Figure S1 — Transcriptional Profiles of LGIT and LGIT Mutant Time Course Infections. (A) As described in Figure 2, LGIT and corresponding Sp1 and κB mutants were infected in Jurkat cells at low MOI (∼0.05–0.10) in biological triplicate, and GFP expression was monitored over a 21-day time course. Histograms from each replicate for each day were used to generate a heat map for days 2–13 of the time course. The heat map indicates the distribution of GFP fluorescence (y-axis) for each LGIT variant and how this distribution changes over time (x-axis). The heat map reflects the GFP fluorescence beyond the autofluorescence threshold, which is set at 2.0 relative fluorescence units (RFU). The depicted region is the sum of “Mid” and “Bright” regions (Figure 1C), and is termed the “On” region. Further details of data analyses are in available in Materials and Methods. Two LGIT variants (mutALL Sp1 and mutIII Sp1/mutI NF-κB) failed to generate a GFP+ population of cells after infection at low MOI (∼0.05–0.10) and were thus omitted from this study (see Figure S1B). (B) LGIT and mutant infections (MOI ∼0.05–0.15) are shown seven days after infection (filled grey). In parallel, each population was stimulated with TNF-α (red outline) or TSA (blue outline) for 18 hours six days after infection. In addition to the previously mentioned Sp1 and κB LGIT mutants, two other mutant combinations (mutation of all Sp1 sites, mutALL Sp1, and mutation of Sp1 site III and κB site I, mutIII Sp1/mutI NF-κB) failed to generate a Bright population after infection and were negligibly responsive to stimulation with TNF-α or TSA. Since infections with these particular LGIT mutants failed to generate a Bright mode, they were omitted from further study, as the focus of this investigation was the molecular and functional differences between Off and Bright modes in a bimodal population. (12 MB TIF) [file ppat.1000260.s001.tif]

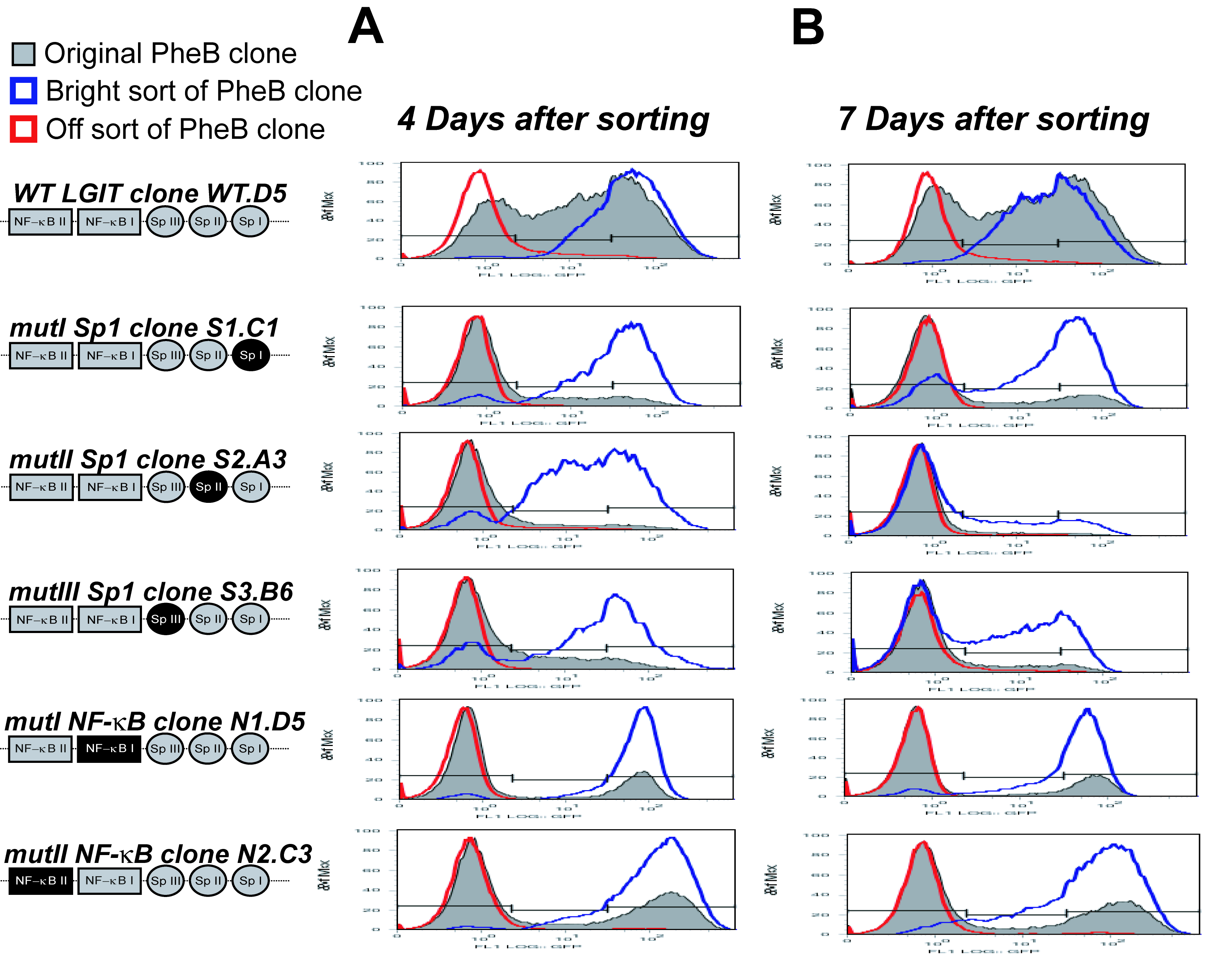

Supplement: Figure S2 — Increased Switching Dynamics for Sp1 Mutant PheB Clones. (A) Transcriptional switching dynamics were measured for one PheB clone for each LGIT variant. Off (red outline) and Bright (blue outline) fractions were isolated with FACS, and flow cytometry was used four days after sorting to measure the degree of switching (see Figure 4D). Off and Bright switching measurements were normalized by the distribution of cells in the unsorted PheB clone (gray filled histogram). Cytometry measurements for the unsorted PheB clones were performed at the same time as the sorts. (B) Same as (A) with flow cytometry measurements performed seven days after sorting. Cytometry measurements for the unsorted PheB clones were performed at the same time as the sorts. Refer to Figure 4E for quantitative results. (2.1 MB TIF) [file ppat.1000260.s002.tif]

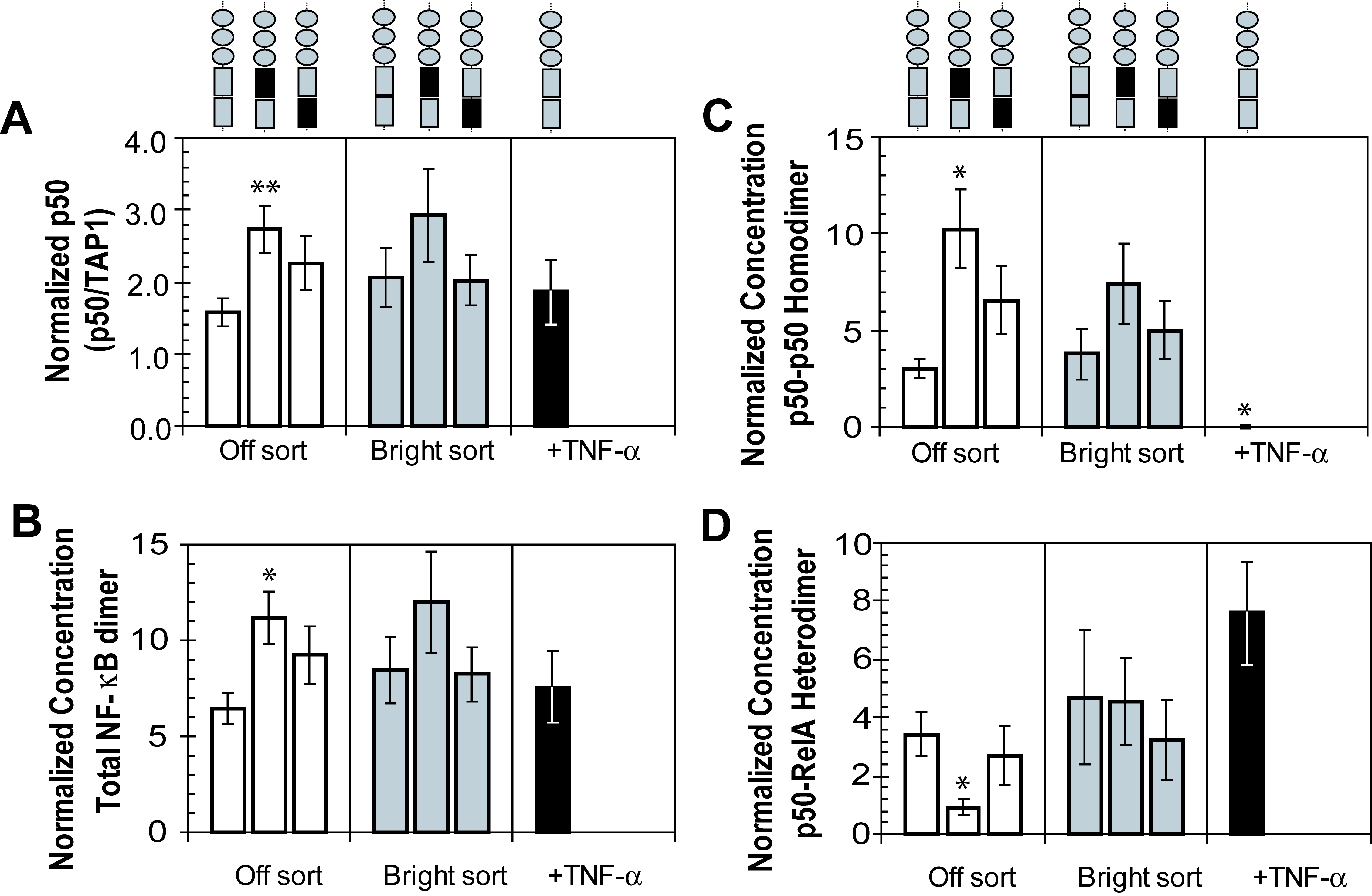

Supplement: Figure S3 — Latent mutI NF-κB Retains Occupancy of p50-p50 Homodimer but not p50-RelA Heterodimer. (A) p50 ChIP results for Off-sorted and Bright-sorted populations of LGIT, mutI NF-κB, and mutII NF-κB as well as TNF-α-stimulated WT LGIT. Immunoprecipitations were performed using p50 antibody, and immunoprecipitated DNA was quantified using QPCR with primers against the HIV LTR. For normalization of input DNA and p50 immunoprecipitation, all LTR QPCR measurements were normalized by with ChIP-QPCR measurements for the endogenous TAP1/LMP2 regulatory domain, which contains single κB and Sp1 sites recruits RelA and p50 (20). Primer sequences and QPCR conditions for HIV LTR and TAP1/LBP2 are supplied in Materials and Methods. The QPCR measurements for LTR and control TAP1/LBP2 were performed in triplicate, and error bars are standard deviations. For all panels in Figure S3, statistically significant differences from the corresponding sort for WT LGIT are denoted by single asterisks (*, p<0.01) and double asterisks (**, p<0.05). (B–D) Calculations of p50-p50 homodimers and p50-RelA heterodimers for Off-sorted and Bright-sorted populations of LGIT, mutI NF-κB and mutII NF-κB and TNF-α-stimulated WT LGIT. WT LGIT was stimulated with TNF-α (20 ng/ml, incubated for 30 minutes before crosslinking), and ChIP was performed using p50 and RelA antibodies. Since TNF-α induces nuclear localization of RelA, for the purposes of an approximate calculation, all NF-κB bound to the κB sites of the LTR can be approximated as p50-RelA heterodimer and none as p50-p50 homodimer. With this assumption, and the supposition that stimulation with TNF-α results in the maximum induced levels of p50 and RelA bound to the HIV LTR, we calculated the maximum normalized concentrations of p50 (p50max = 1.86) and RelA (RelAmax = 7.6). The ratio of induced measurements of RelA to p50 (7.6/1.86 = 4.1) corresponds to the relative efficiency in RelA and p50 immunoprecipitations. Let the variables x and y be the concentra [file ppat.1000260.s003.tif]

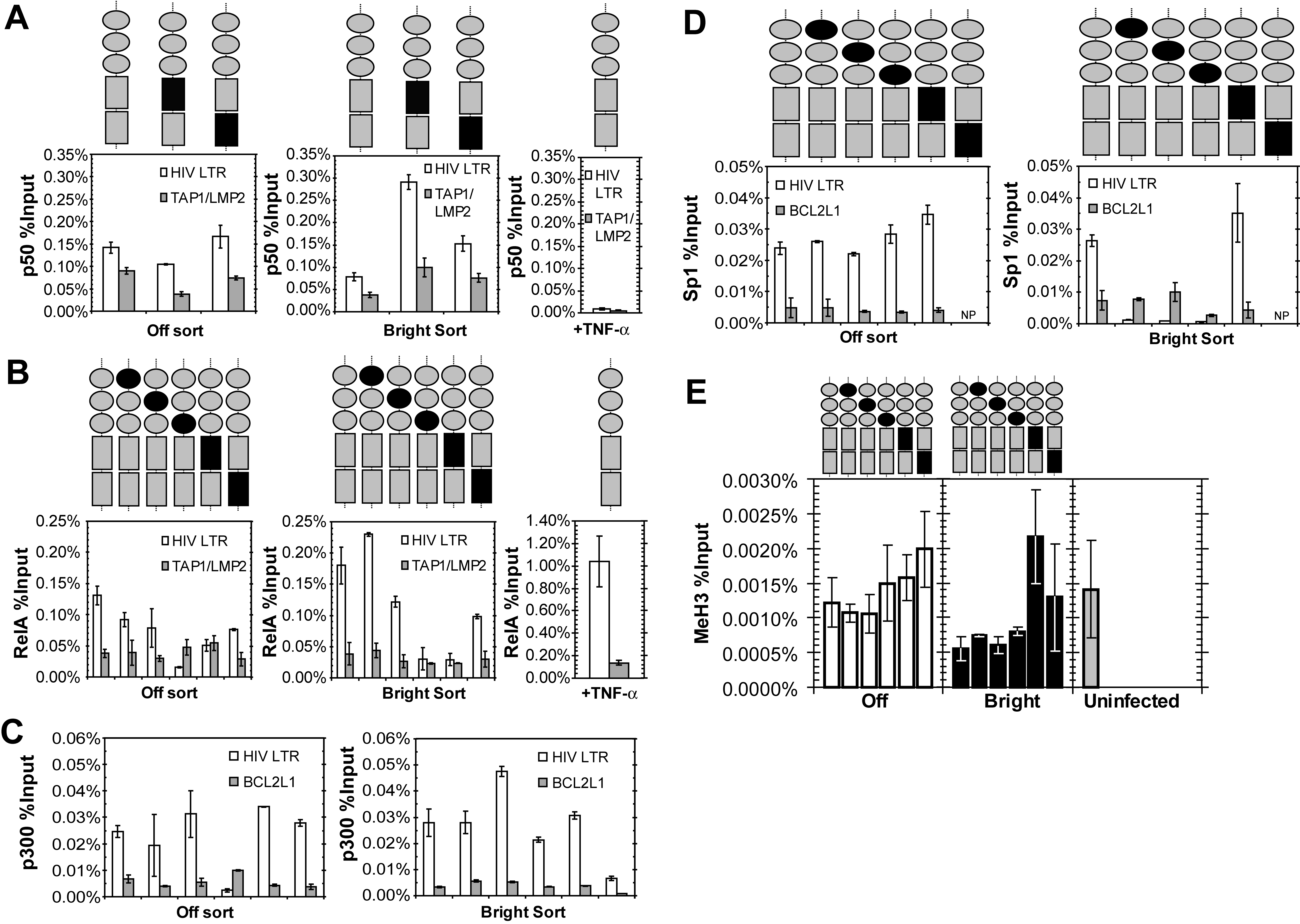

Supplement: Figure S4 — Non-normalized ChIP QPCR as a Percentage of Input DNA. (A) As performed in Figure S3, ChIP QPCR results as a percentage of input against p50 for Off- and Bright-sorted LGIT, mutI NF-κB, and mutII NF-κB and TNF-α-induced WT LGIT. Denoted are the HIV LTR (white bars) and the control gene, TAP1/LBP2 (gray bars). mutI Sp1, mutII Sp1, and mutIII Sp1 were not performed. (B) As performed in Figure 6B, ChIP QPCR results as a percentage of input DNA against RelA for Off- and Bright-sorted LGIT, mutI Sp1, mutII Sp1, mutIII Sp1, mutI NF-κB, and mutII NF-κB and TNF-α-induced WT LGIT. Denoted are the HIV LTR (white bars) and the control gene, TAP1/LMP2 (gray bars). This is the same control gene as used for p50. (C) As performed in Figure 6C, ChIP QPCR results as a percentage of input against p300 for Off- and Bright-sorted LGIT, mutI Sp1, mutII Sp1, mutIII Sp1, mutI NF-κB, and mutII NF-κB. Denoted are the HIV LTR (white bars) and the control gene, BCL2L1 (gray bars). (D) As performed in Figure 6D, ChIP QPCR results as a percentage of input against Sp1 for Off- and Bright-sorted LGIT, mutI Sp1, mutII Sp1, mutIII Sp1, and mutI NF-κB. Denoted are the HIV LTR (white bars) and the control gene, BCL2L1 (gray bars). This is the same control gene as used for p300 and mutII NF-κB, denoted as “NP”, was not performed. (E) ChIP QPCR results as a percentage of input against trimethylated histone 3 lysine 9 (TriMetH3K9) for Off- and Bright-sorted LGIT, mutI Sp1, mutII Sp1, mutIII Sp1, mutI NF-κB, and mutII NF-κB. Off-sorts are denoted by white bars, Bright-sorts are denoted by black bars, and uninfected Jurkat is denoted by the gray bar. Note that no samples exceed the background (uninfected) control, indicating that TriMetH3K9 is below detection levels. (1.8 MB TIF) [file ppat.1000260.s004.tif]

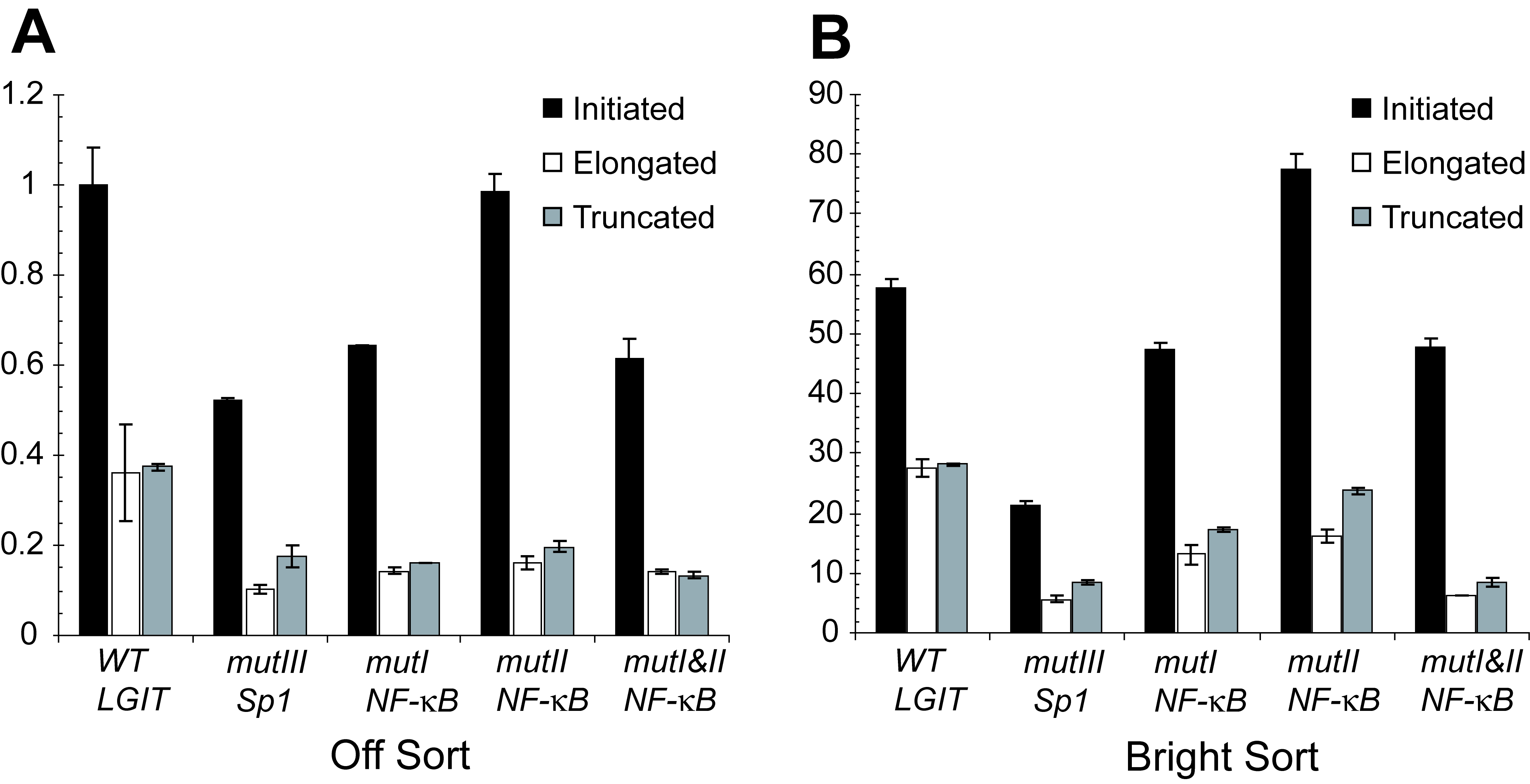

Supplement: Figure S5 — Calculations of Truncated Transcripts by RT-PCR. (A–B) As performed in Figures 6G–H, real time RT-PCR analysis on initiated and fully elongated transcripts for Off- and Bright-sorted LGIT, mutIII Sp1, mutI NF-κB, mutII NF-κB, and mutI&II NF-κB. Off and Bright sorts were performed as in Figure 6A, and cells were expanded for approximately one week before mRNA extraction. Details for mRNA preparation QPCR are in Materials and Methods in the main text. Initiated transcripts were detected with primers for TAR, and elongated transcripts were detected with primers for Tat. For both panels, statistically significant differences from the corresponding sort for WT LGIT are denoted by single asterisks (*, p<0.01) and double asterisks (**, p<0.05). (0.92 MB TIF) [file ppat.1000260.s005.tif]

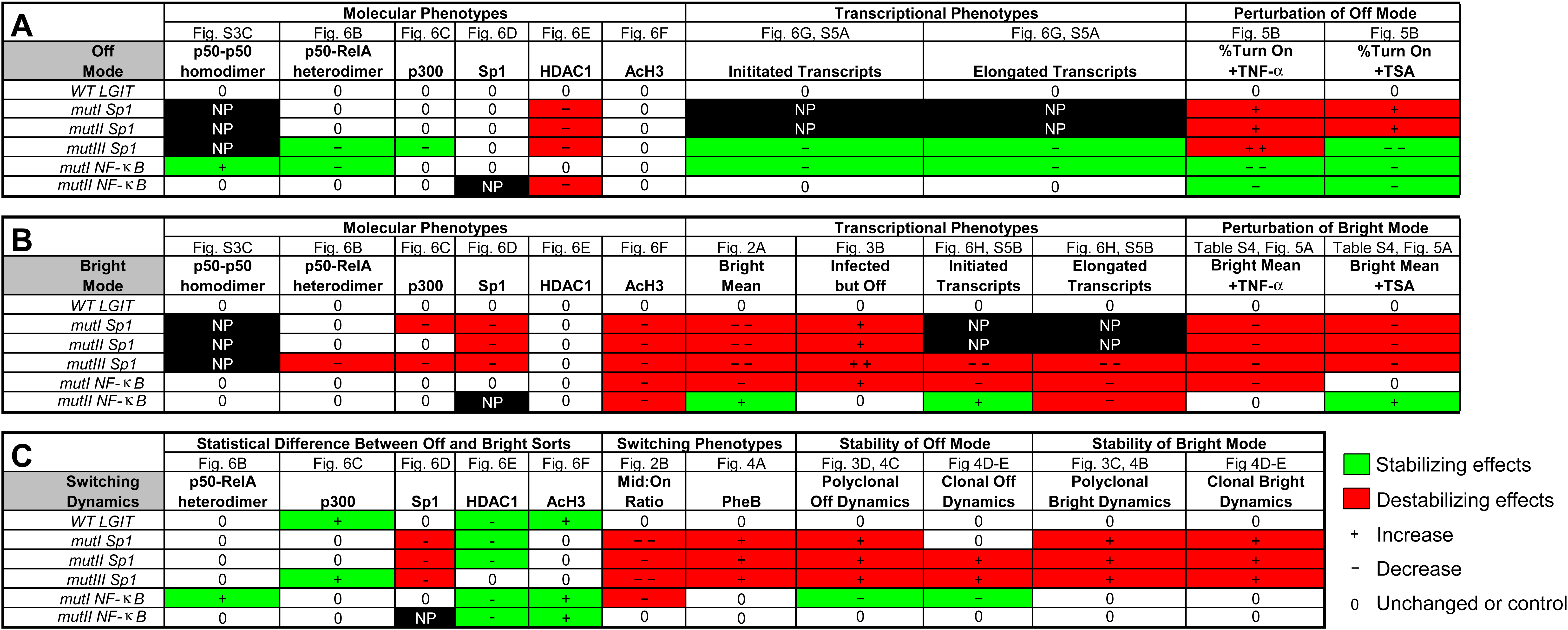

Supplement: Figure S6 — Stabilizing and Destabilizing Phenotypes for Off and Bright Modes. (A) Summary of molecular and transcriptional phenotypes in the inactive (Off) transcriptional mode. Measurements for WT LGIT are assigned a “0” value, and comparisons for each mutant to WT LGIT are denoted by increases (+), decreases (−), and unchanged (0) values. Potential changes to the stabilities of the Off mode are indicated by green (stabilizing) and red (destabilizing) boxes. Measurements denoted with “NP” (black boxes) were not performed. (B) Same as in (A) for the active (Bright) transcriptional mode. Potential changes to the stabilities of the Bright mode are indicated by green (stabilizing) and red (destabilizing) boxes. Measurements denoted with “NP” (black boxes) were not performed. (C) Differential regulation of the Off and Bright modes was determined by examining phenotypic differences in the Off- and Bright-sorts and dynamic switching between modes. For ChIP measurements, statistically significant increases (+), decreases (−), and unchanged (0) values for Bright sorts, compared to the Off-sorted fractions, are denoted. For switching and dynamics phenotypes, mutant values are compared to the WT LGIT counterpart, as in (A) and (B). Differential regulation of Off and Bright modes is indicated by green (strongly regulated) and red (disregulated) boxes. Measurements denoted with “NP” (black boxes) were not performed. (1.5 MB TIF) [file ppat.1000260.s006.tif]
