# Supplementary material for: Control of Stochastic Gene Expression by Host Factors at the HIV Promoter
Source: PLoS Pathog. 2009 Jan 9;5(1):e1000260. doi: 10.1371/journal.ppat.1000260 (PMC2607019; doi:10.1371/journal.ppat.1000260)
Supplement: Table S1 — Quikchange Primer Sequences for LGIT mutants (0.04 MB DOC) [file ppat.1000260.s007.doc]

| Forward mutI Sp1: | gggcgggactgg**TT**agtggcgagccc |
| --- | --- |
| Reverse mutI Sp1: | GGGCTCGCCACT**AA**CCAGTCCCGCCC |
| Forward mutII Sp1: | gggaggcgtggcctg**TT**cgggactggggagtgg |
| Reverse mutII Sp1: | CCACTCCCCAGTCCCG**AA**CAGGCCACGCCTCCC |
| Forward mutIII Sp1: | gggactttccagggA**TT**Cgtggcctggg |
| Reverse mutIII Sp1: | CCCAGGCCACG**AA**TCCCTGGAAAGTCCC |
| Forward mutI NF-κB: | ggactttccgctg**tct**actttccagg |
| Reverse mutI NF-κB: | CCTGGAAAGT**AGA**CAGCGGAAAGTCC |
| Forward mutII NF-κB: | gcttgctacaa**tct**actttccgctgg |
| Reverse mutII NF-κB: | CCAGCGGAAAGT**AGA**TTGTAGCAAGC |
| Forward mut I&II NF-κB: | gcttgctacaa**tct**actttccgctg**tct**actttccagg |
| Reverse mut I&II NF-κB: | CCTGGAAAGT**AGA**CAGCGGAAAGT**AGA**TTGTAGCAAGC |
| Forward delNF-κB: | AACTGCTGACATCGAGCTTGCTACAGGGAGGCGTGGCCTGGGCG |
| Reverse delNF-κB: | GCCCAGGCCACGCCTCCCTGTAGCAAGCTCGATGTCAGCAGTTC |
| Forward mutALL Sp1: | GGACTTTCCAGGGA**TT**CGTGGCCTG**TT**CGGGACTGG**TT**AGTGGCGAGC |
| Reverse mutALL Sp1: | GCTCGCCACT**AA**CCAGTCCCG**AA**CAGGCCACG**AA**TCCCTGGAAAGTCC |
