# Supplementary material for: Control of Stochastic Gene Expression by Host Factors at the HIV Promoter
Source: PLoS Pathog. 2009 Jan 9;5(1):e1000260. doi: 10.1371/journal.ppat.1000260 (PMC2607019; doi:10.1371/journal.ppat.1000260)
Supplement: Table S2 — Primer Sequences for ChIP QPCR (0.03 MB DOC) [file ppat.1000260.s008.doc]

| **ChIP Primers for QPCR of HIV-1 LTR** (Williams, 2004)  (201 bp amplicon, PubMed accession# AF033819.3)  LTRκB-primer5: AGGTTTGACAGCCGCCTA  LTRκB-primer3: AGAGACCCAGTACAGGCAAAA |
| --- |
| **ChIP Primers for QPCR of *TAP1/LMP2* (p50 and RelA control)** (Wright, 1995)  (249 bp amplicon, PubMed accession# NM_000593.5)  5′TAP1/LMP2: CCAGGGCTGCTCCCGAGATTCTCAG  3′TAP1/LMP2: CCTGAAGCTCCGGGTACCGCCGAG |
| **ChIP Primers for QPCR of *BCL2L1* (Sp1 and p300 control)** (Smith, 2004)  (173 bp amplicon, PubMed accession# NW_001838664.2)  5′BCL2L1: ACCAACTAAATCCATACCAGCCACCTCC  3′BCL2L1: CCCCTCGCTTGCTTCCTCCTCC |
