# Supplementary material for: Control of Stochastic Gene Expression by Host Factors at the HIV Promoter
Source: PLoS Pathog. 2009 Jan 9;5(1):e1000260. doi: 10.1371/journal.ppat.1000260 (PMC2607019; doi:10.1371/journal.ppat.1000260)
Supplement: Table S3 — Primer Sequences for Quantitect RT-PCR (0.03 MB DOC) [file ppat.1000260.s009.doc]

| **RT-PCR Primers for TAR (total HIV-1 transcripts) (**Williams, 2006)  HIVTAR5: GTTAGACCAGATCTGAGCCT  HIVTAR3: GTGGGTTCCCTAGTTAGCCA |
| --- |
| **RT-PCR Primers for TAR (elongated HIV-1 transcripts)**  Tat5: GCAGGAAGAAGCGGAGAC  Tat3: CTTGATGAGTCTGACTGCCTTG |
| **RT-PCR Primers for β-Actin** (Weinberger, 2005)  β-Actin5: ACCTGACTGACTACCTCATGAAGATCCTCACCGA  β-Actin3: GGAGCTGGAAGCAGCCGTGGCCATCTCTTGCTCGAA |
