# Supplementary material for: Control of Stochastic Gene Expression by Host Factors at the HIV Promoter
Source: PLoS Pathog. 2009 Jan 9;5(1):e1000260. doi: 10.1371/journal.ppat.1000260 (PMC2607019; doi:10.1371/journal.ppat.1000260)
Supplement: Table S4 — Non-normalized Bright Mean Position (RFU) (0.03 MB DOC) [file ppat.1000260.s010.doc]

|  | **Unperturbed** | **TNF-α** | **TSA** |
| --- | --- | --- | --- |
| *WT* | 301 (RFU) | 585 | 541 |
| *mutI Sp1* | 145 | 339 | 273 |
| *mutII Sp1* | 176 | 346 | 331 |
| *mutIII Sp1* | 135 | 327 | 151 |
| *mutI NF-κB* | 243 | 382 | 490 |
| *mutII NF-κB* | 313 | 553 | 567 |
| *mutI&II NF-κB* | 274 | 278 | 443 |
